# Kisspeptin Mitigates Hepatic De Novo Lipogenesis in Metabolic Dysfunction-Associated Steatotic Liver Disease

**DOI:** 10.3390/cells14161289

**Published:** 2025-08-20

**Authors:** Kimberly Izarraras, Ankit Shah, Kavita Prasad, Helena Tan, Zhongren Zhou, Moshmi Bhattacharya

**Affiliations:** 1 Department of Medicine, Robert Wood Johnson Medical School, Rutgers University, New Brunswick, NJ 08901, USA; ki113@gsbs.rutgers.edu (K.I.); ashah386@rwjms.rutgers.edu (A.S.); kp460@connect.rutgers.edu (K.P.); hyt9@scarletmail.rutgers.edu (H.T.); 2 Department of Pathology and Laboratory Medicine, Robert Wood Johnson Medical School, Rutgers University, New Brunswick, NJ 08901, USA; zz442@rwjms.rutgers.edu

**Keywords:** kisspeptin, KISS1R, liver, steatosis, MASLD, de novo lipogenesis, CIDEA, SREBP

## Abstract

The peptide hormone kisspeptin, signaling via its receptor, KISS1R, decreases hepatic steatosis and protects against metabolic dysfunction-associated steatotic liver disease (MASLD). Enhanced de novo lipogenesis (DNL) contributes to MASLD. Here, we investigated whether kisspeptin treatment in obese, diabetic mice directly attenuates DNL. DNL was assessed in kisspeptin-treated mouse livers, using a mouse model of MASLD, (DIAMOND mice), employing ^2^H_2_O-enriched water, mass spectrometry analysis, and transcriptomic profiling. Gene and protein expression were evaluated in primary hepatocytes and livers. Additionally, hepatic *Kiss1r* expression was increased in DIAMOND mice, following which various biochemical and metabolic assessments were employed. Metabolic tracing in kisspeptin-treated steatotic livers demonstrated a decrease in the DNL of free fatty acids (FFAs), known to be associated with diabetes, steatosis, and hepatocellular carcinoma. Transcriptomic profiling of kisspeptin-treated livers identified disruption of key metabolic pathways, the most prominent being a decrease in fatty acid metabolism, and downregulation of *Cidea*, a key regulator of lipid droplet formation. Kisspeptin treatment of FFA-loaded primary mouse hepatocytes significantly decreased *Cidea* expression. Mechanistically, we found that kisspeptin administration decreased levels of transcription factor SREBP-1c, a crucial regulator of DNL, and CIDEA. Thus, enhanced KISS1R signaling limits hepatic DNL, suggesting a crucial role in restricting MASLD.

## 1. Introduction

Metabolic dysfunction-associated steatotic liver disease (MASLD), formerly called nonalcoholic fatty liver disease (NAFLD), is the most widespread chronic liver disease, with a 30% global prevalence [1]. The rise in MASLD parallels the increase in other metabolic diseases, including obesity and type 2 diabetes. A defining characteristic of MASLD is steatosis, which is characterized by an excess of hepatic lipids, that can give rise to a spectrum of liver histopathologies including steatohepatitis, fibrosis/cirrhosis, and hepatocellular carcinoma. Hepatic steatosis stems from increased hepatic de novo lipogenesis (DNL), which is the synthesis of fatty acid chains from acetyl-CoA; these fatty acid chains undergo an esterification reaction with a glycerol for triglyceride synthesis [2]. Cirrhosis and hepatocellular carcinoma lead to increased morbidity and mortality and an increased demand for liver transplantation among those affected [3,4]. Given the substantial healthcare burden posed by MASLD, effective therapies are urgently needed, particularly therapies aimed at reducing the underlying increase in DNL.

Kisspeptin, encoded by the *KISS1* gene, is a circulating peptide hormone that signals via the G protein-coupled receptor, kisspeptin 1 receptor (KISS1R) [5,6]. The liver produces kisspeptin [7]. We previously showed that the KISS1/KISS1R signaling pathway protects against MASLD development in mouse models [8]. Depletion of hepatic *Kiss1r* exacerbated hepatic steatosis, while the pharmacological enhancement of KISS1R signaling lowered hepatic triglyceride content, serum triglyceride levels, and serum free fatty acid levels [8]. Mechanistically, we found that KISS1R signaling activates the energy sensor AMP-activated protein kinase (AMPK), leading to enhanced mitochondrial fatty acid oxidation in the liver. Thus, based on the accumulating evidence demonstrating a protective effect of KISS1R signaling in the development of MASLD, we hypothesize that KISS1R signaling directly decreases DNL and the formation of new intrahepatic triglycerides. In this study, we investigated whether kisspeptin signaling regulates the DNL of free fatty acids using a deuterium water tracer. We also investigated the impact of overexpressing hepatic *Kiss1r* on steatosis. These studies reveal a new mechanism of action, by which KISS1R signaling limits hepatic DNL.

## 2. Materials and Methods

### 2.1. Animal Studies

**Figure 1:**
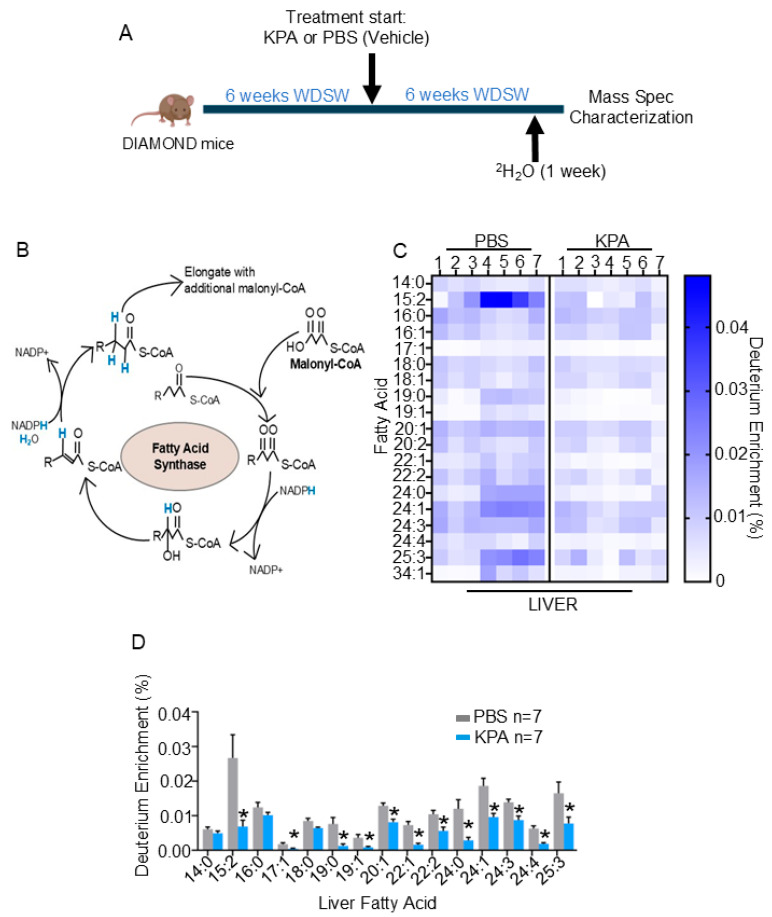
KPA (kisspeptin analog) treatment decreases hepatic de novo lipogenesis. (**A**) Study timeline: DIAMOND mice fed a WDSW were treated with either KPA (kisspeptin analog) or PBS (Vehicle); (N = 7/cohort). ^2^H_2_O-enriched water was given for 7 days prior to euthanasia (**B**) Schematic showing incorporation of deuterium from ^2^H_2_O-enriched water into newly synthesized fatty acids (blue) and elongated fatty acid chains during hepatic de novo lipogenesis. (**C**) Heatmap of deuterium enrichment in intrahepatic fatty acids in KPA and control livers: C14:0 (Myristic acid); C15:2 (Pentadecadienoic acid); C16:0 (Palmitic acid); C16:1 (Palmitoleic acid); C17:1 (Heptadecenoic acid); C18:0 (Steric acid); C18:1 (Oleic acid); C19:0 (Nonadecanoic acid); C19:1 (Nonadecanoic acid); C20:1 (Eicosenoic acid); C20:2 (Eicosadienoic acid); C22:1 (Erucic acid); C22:2 (Docosadienoic acid); C24:0 (Docosadienoic acid); C24:1 (Tetracosenoic acid); C24:3 (Tetracosatrienoic acid); C24:4 (Tetracosatetraenoic acid); C25:3 (Pentacosatrienoic acid); C34:1 (Tetratriacontenoic acid). (**D**) Deuterium enrichment of individual intrahepatic fatty acids. Results expressed as mean ± S.E.M. Student’s unpaired *t*-test, * *p* < 0.05 versus respective controls.

All animal procedures were approved by the Rutgers University Institutional Animal Care and Use Committee (PROTO201702536) in accordance with Institutional Animal Care and Use Committee guidelines. Mice were housed in a pathogen-free barrier facility and maintained on a 12 h light/dark cycle. Since there is a higher prevalence of MASLD in males than females [9]; these initial investigations studying the effect of kisspeptin on DNL were conducted using male mice. Diet-Induced Animal Model of Non-Alcoholic Liver Disease (DIAMOND) mice were obtained from Sanyal Biotechnologies [10]; this is an isogenic strain obtained from C57BL/6J & 129S1/Svlm. DIAMOND mice (8 weeks old) were subsequently placed on a high-fat ‘Western’ diet (42% calories from 40 fat, 0.2% from cholesterol, 42.7% calories from carbohydrate, 15.2% calories from protein; Diet # TD.88137) and sugar water (23.1 g/L of fructose and 18.9 g/L of glucose) to recapitulate human MASLD [10,11]. The following experiments were conducted using independent cohorts of mice, randomly assigned to groups for allocation, and included littermate controls. Additionally, experiments and analysis were performed blindly. Based on our previous data using mouse models of MASLD [8], power analysis calculations were conducted to detect a 25% difference in pathologic metrics defined above with a power of 0.8 and *p* < 0.05. Mice (7/group) were maintained on Western diet/sugar water (WDSW) for 6 weeks to induce steatosis, and then randomly administered a stable, long-acting KP-analog, TAK-448 (referred to as KPA, 0.3 nmol/h) purchased from MedChemExpress (Monmouth Junction, NJ, USA) or vehicle (phosphate-buffered saline, PBS), using Alzet mini-osmotic pump model 2004 (Durect, Cupertino, CA, USA) as previously described [8]. KPA and PBS were administered for 6 weeks, while mice were maintained on WDSW (Figure 1A). Various metabolic tests were conducted on these mice. A week before euthanization, after 5 weeks of treatment, drinking water was replaced with 20% ^2^H_2_O-enriched water to quantify deuterium incorporation into newly synthesized fatty acids, as described [12]. Animals were euthanized by CO_2_ or cervical dislocation, and tissue was collected for analysis, blindly (Figure 1A). Although not common, mice that developed an infection or whose pumps were removed due to fighting were excluded from the study.

For overexpression of hepatic *Kiss1r*, male DIAMOND mice (8 weeks of age, 5/group) were placed on WDSW for 12 weeks and then randomly injected via the tail vein with either AAV8-TBG-m-KISS1R (NM_053244, Vector Biolab, Malvern, PA, USA) or Control AAV8-TBG-eGFP (VB1743, Vector Biolab; 5 × 10^11^ genome copies/mouse). After another 6 weeks on WDSW, mice underwent metabolic testing, including blinded assessment using the Comprehensive Laboratory Animal Monitoring System (CLAMS), prior to euthanasia as described [8]; off-target effects were not observed. KPA-treated high-fat-fed, hepatic deletion of AMPK (shAMPK) has been previously characterized [8].

### 2.2. Deuterium Labeling and Metabolomics

These experiments were performed as described [13]. DIAMOND mice on WDSW were given drinking water with 20% ^2^H_2_O-enriched water (Cambridge Isotope Laboratories, Andover, MA, USA) for seven days before tissue harvesting (Figure 1A). To measure deuterium enrichment of intrahepatic fatty acids, liver tissues were processed as previously described [13]. Briefly, hepatic tissues (~10 mg) were homogenized with precooled methanol (12 µL/mg of tissue) and then mixed with 20 °C methyl tert-butyl ether (40 μL/mg of tissue). The samples were then shaken for six minutes at 4 °C, followed by the addition of H_2_O (10 μL/mg of tissue), and centrifuged at 13,000× *g* for two minutes. The resulting top lipid layer was isolated, dried under air, and resuspended in 1 mL of saponification solvent (0.3 mM KOH in 90:10 methanol:H_2_O), and incubated in an 80 °C water bath for one hour. Afterward, samples were cooled on ice for three minutes and then vortexed in a solution of 100 μL of formic acid and 300 μL of hexanes. The resulting top layer was transferred to a new tube, and the step was repeated once to obtain a total of 600 mL. This subsequent extract was air-died and resuspended in 150 mL of 50:50 isopropanol:methanol solution. All reagents were of LC-MS grade (Thermo Fisher Scientific, Waltham, MA, USA). Samples were analyzed by liquid chromatography-mass spectrometry (LC-MS) under positive polarity on Q Exactive Plus mass spectrometer coupled with a Vanquish Horizon UHPLC system (Thermo Fischer Scientific, Waltham, MA, USA) using a Poroshell 120 EC-C18 column (150 mm × 2.1 mm, 2.7 µm particle size, Agilent InfinityLab, Santa Clara, CA, USA). Data were analyzed blindly using El-MAVEN software v0.12.0 suite [14]. The natural isotope abundances were corrected using R package AccuCor v0.2.3 [15].

### 2.3. Metabolic Tests and Comprehensive Lab Animal Monitoring System (CLAMS)

These studies were analyzed blindly and performed as described [8]. Blood glucose levels were measured with a glucometer (Bayer Contour, Ascensia, Parsippany, NJ, USA). For the glucose tolerance test (GTT), mice were fasted for 12 h, and D-glucose (1 g/kg; Sigma-Aldrich, Allentown, PA, USA) was injected intraperitoneally before conducting glucose measurements. This test was performed after nine weeks on WDSW, and after three weeks of treatment. For the insulin tolerance tests (ITT), mice were fasted for 6 h, and insulin (1 U/kg; Novo Nordisk, Plainsboro, NJ, USA) was injected intraperitoneally before conducting glucose measurements. ITT was performed after ten weeks on WDSW and after four weeks of treatment. For measuring respiration, energy expenditure, ambulatory movement, and feeding, mice were individually placed in CLAMS with controlled lighting and feeding; recordings were conducted over four days, before mice were euthanized.

### 2.4. Quantitative Real-Time PCR

These experiments were performed as described [8,16]. To isolate RNA, TRIzol reagent (ThermoFisher, Waltham, MA, USA) was added to mouse tissues or mouse hepatocytes followed by RNeasy (Qiagen, Valencia, CA, USA) to isolate RNA. Reverse transcription was then performed using iScript RT Supermix (Bio-Rad, Hercules, CA, USA), according to the manufacturer’s instructions. SYBR green real-time qPCR (RT-qPCR) was used to assess gene expression as described previously [8,16] using primers (Supplemental Table S1). Two-step RT-qPCR was used and gene expression analyzed using the 2^−ΔΔCt^ analysis method.

### 2.5. Immunoblot Analysis

These experiments were performed as described [8,16]. RIPA lysis buffer containing protease and phosphatase inhibitors was used for preparing mouse liver lysates, and protein expression was quantified using the Lowry Method. Western blot analysis was performed using 100 μg of protein per lane using sodium dodecyl sulfate polyacrylamide del electrophoresis (SDS-PAGE,) and the antibodies (Supplemental Table S2). The gels were transferred using semi-dry transfer onto nitrocellulose membrane (Bio-Rad). Nuclei isolation was performed using PARIS Kit (ThermoFisher, Waltham, MA, USA, Catalog # AM1921), according to the manufacturer’s instructions. Blots were imaged using Super Signal West Dura Extended Duration Substrate (Thermo Scientific, Waltham, MA, USA, Catalog # 34076) and ChemiDoc Touch imaging system (Bio-Rad, Hercules, CA, USA). Protein expression was quantified using Image Lab Software (Bio-Rad, Version 6.1.0 build 7, Standard Edition).

### 2.6. Alanine Aminotransferase (ALT), Free Fatty Acids (FFA), and Triglycerides Measurements

All assays were performed according to the manufacturer’s instructions. ALT levels were measured using ALT Activity Assay (Millipore Sigma, Saint Louis, MO, USA, Catalog # MAK052-1KT). Serum and liver triglycerides were measured using the triglyceride quantification kit (Millipore Sigma, Catalog # MAK266-1KT). Free fatty acids were measured using the Free Fatty Acid Quantification Kit (Millipore Sigma, Catalog # MAK044-1KT).

### 2.7. RNA Sequencing

RNA sequencing was conducted as previously described [16]. Total RNA isolated from DIAMOND mice livers was quantified by Qubit (Invitrogen: Thermo Fisher Scientific) at Albert Einstein College of Medicine Epigenomics Shared Facility (ESF), and RNA quality was assessed by using the 4150 TapeStation System (Agilent Technologies, Santa Clara, CA, USA). Purified total RNA was used to prepare Libraries following the protocol using Qiaseq Stranded RNA lib Kit with UDI and QIAseq FastSelect-rRNA HMR Kit (Qiagen Inc.) for Illumina sequencing. Libraries were QC using Fluorometric Quantification (Qubit; Invitrogen: Thermo Fisher Scientific), Aligent 4150 TapeStation System and QPCR (Roche Light Cycler, San Francisco, CA, USA). RNASeq libraries were multiplexed and sequenced as 1 × 100 bp single end on NEXTSEQ 2000 (Illumina, San Diego, CA, USA) following standard protocols. The sequencing files were aligned with STAR v2.7.9a and the STAR software itself generated the read counts; the relative counts of sequences from each gene relative to each other (transcriptional profiling), normalization and statistical comparison was then determined. Differential gene analyses were performed using the R package DESeq2 v1.36.0. The genes with more than a 2-fold change and adjusted *p*-values below 0.05 were considered significantly different. Gene set enrichment analysis was conducted using the R package v1.28.0 against KEGG and the GO database.

### 2.8. Isolation of Primary Hepatocytes

Primary hepatocytes were isolated from mice livers as described [8]. Livers were cannulated via the hepatic portal vein and perfused with Kreb’s Ringer solution containing EGTA, and then Kreb’s Ringer solution containing CaCl_2_ and Liberase™ Roche (Millipore Sigma, Catalog # 5401119001), to dissociate the tissue. The dissociated solution was filtered, and cells were then resuspended in William’s Media E (Sigma-Aldrich, Allentown, PA, USA) with 10% fetal bovine serum (Sigma-Aldrich, Allentown, PA, USA), 200 nM dexamethasone (Sigma-Aldrich, Allentown, PA, USA), penicillin-streptomycin (10,000 U/mL, Thermo Fisher Scientific, Waltham, MA, USA) and 2 mM L-glutamine (Thermo Fisher Scientific, Waltham, MA). Hepatocytes (3 × 10^5^ cells) were cultured on 6-well collagen (Sigma-Aldrich, Allentown, PA, USA) coated plates. Free fatty acids (150 μM oleic acid and 150 μM palmitic acid) was conjugated to 2% BSA. Cells were cultured overnight with FFAs, followed by serum starvation for 3 h and then treated with KPA or vehicle (PBS) for 24 h.

### 2.9. Immunohistochemistry

Histological processing was performed by the Research Pathology Services at Rutgers University. Oil Red O (ORO) staining was performed on cryostat sections of the frozen liver tissues to identify neutral lipids and quantified using QuPath an open-source software (GNU General Public License v3.0). Immunohistochemical analysis of mouse livers, hematoxylin and eosin (Vector Laboratory, Newark, CA, USA, H&E Kit, Catalog # H3502) and ORO staining (Abcam, Waltham, MA, USA, Oil Red O Stain Kit (Lipid Stain), Catalog # 150678) was reviewed and assessed blindly by the pathologist, Dr. Zhou.

### 2.10. Statistical Analysis

The data are presented as mean ± SEM. Deuterium enrichment data were calculated as atom percent excess with the formula [(^2^H)/(^1^H + ^2^H)] × 100. The area under the curve (AUC) was calculated using the trapezoidal method [17]. A two-tailed Student *t*-test was used to compare the differences between the two groups. A *p*-value of <0.05 was considered to be statistically significant. Graphs and statistical analyses were generated with Graphpad Prism version 10 (GraphPad Software, Inc., San Diego, CA, USA).

## 3. Results

### 3.1. KISS1R Agonist Alleviates Hepatic De Novo Lipogenesis Assessed Using Lipogenic Flux Measurement

The synthesis of fatty acids through DNL is significantly elevated in patients with steatosis [18]. To test our hypothesis that kisspeptin signaling directly regulates hepatic DNL, we conducted a metabolic tracing experiment using deuterated (^2^H_2_O-enriched) water to label de novo-synthesized fatty acids in vivo [19]. DIAMOND mice were placed on a Western diet and sugar water (WDSW) for six weeks, and a kisspeptin analog, KP analog, TAK-448 (0.3 nmol/h, referred to as KPA) [8], was administered for an additional six weeks. During the final week of being maintained on WDSW and KPA infusion, DIAMOND mice were given drinking water containing 20% ^2^H_2_O (Figure 1A). Livers were then collected, processed, and analyzed by mass spectrometry for deuterium enrichment, a measure of DNL. ^2^H_2_O labels lipogenic fat directly and via NADPH (Figure 1B). KPA significantly reduced intra-hepatic deuterium enrichment of long-chain fatty acid compared to controls (Figure 1C,D). Several of these fatty acids such as C15:2, C17:1, C19:0, C19:1 and C22:2 are associated with obesity, type 2 diabetes and steatosis [20,21] (Figure 1D). A significant reduction in deuterium enrichment was also observed in fatty acid species associated with inflammation and hepatocellular carcinoma (HCC) such as eicasonoid (C20:1) and very long chain fatty acids such C22:1, C24:3 and C24:4 [22] (Figure 1D). While not significant, there was also a trend towards a decrease in fatty acids such as C16:0 and C18:0, upon treatment (Figure 1D).

**Figure 2:**
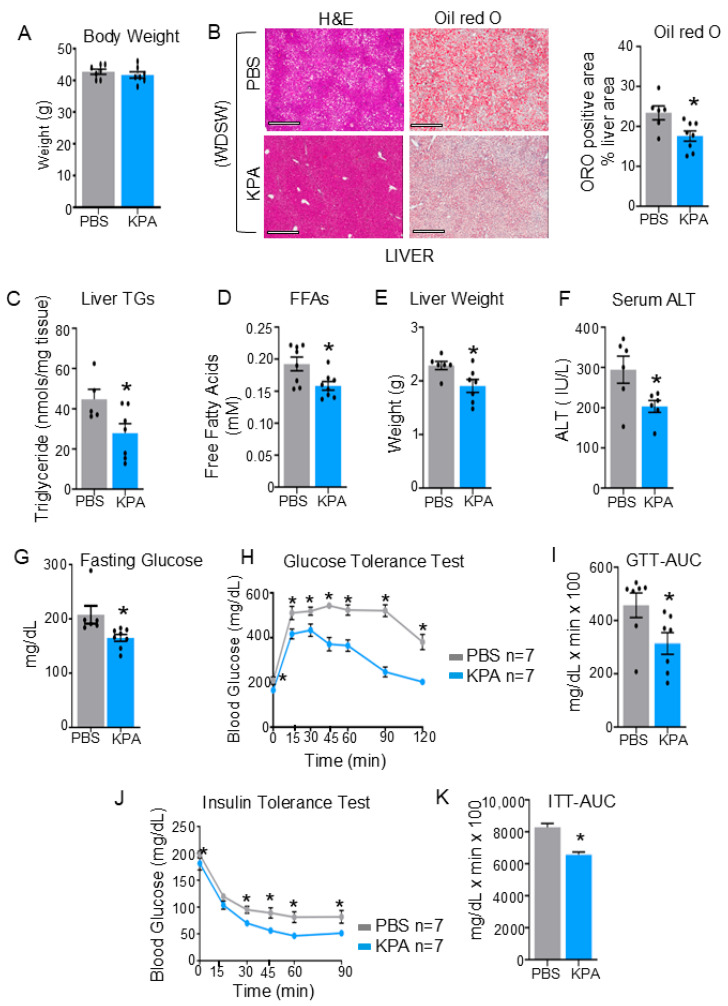
KPA administration protects against hepatic steatosis and improves insulin resistance in DIAMOND mice. DIAMOND mice (N = 7/group, see Figure 1A) on a WDSW diet for 12 weeks were given KPA or PBS (control) for 6 weeks. Following euthanization, the following measurements were taken: (**A**) Body weight. (**B**) Assessment of hepatic lipid content: representative hematoxylin and eosin (H&E) (left) and Oil Red (ORO) stained (right) liver sections; quantification of ORO was performed blindly by a pathologist. Scale bars: 400 μm. (**C**) Liver triglycerides (TGs), (**D**) serum free fatty acids (FFA) levels, (**E**) liver weight, and (**F**) serum alanine aminotransferase (ALT). Metabolic tests: (**G**) fasting blood glucose levels (**H**) a glucose tolerance test (GTT); (**I**) area under the curve (AUC) for GTT. (**J**) Blood glucose levels measured during insulin tolerance test (ITT) and (**K**) area under the curve for ITT. Results expressed as mean ± S.E.M. Student’s unpaired *t*-test; one-way ANOVA followed by Dunnett’s post hoc test. * *p* < 0.05 versus respective control.

KPA treatment or administration of deuterated water did not alter body weight (Figure 2A). Similarly to our previous observations on the effect of KPA on steatosis [8], in this experimental model given deuterated water, KPA treatment significantly reduced hepatic steatosis, as assessed using Oil Red O staining that marks hepatic lipids (Figure 2B), decreased liver triglyceride content (Figure 2C), and reduced circulating levels of free fatty acids (Figure 2D). Additionally, KPA treatment lowered liver weight (Figure 2E), and serum ALT levels (Figure 2F), a marker for liver disease. Since MASLD is associated with insulin resistance, we examined the effects of KPA treatment on glucose homeostasis. KPA improved fasting glucose levels, suggesting a decrease in hepatic glucose production, and increased glucose tolerance, in addition to decreasing insulin resistance (Figure 2G–K), compared to controls.

Taken together, these results demonstrate for the first time that pharmacological enhancement of kisspeptin signaling downregulates hepatic DNL.

### 3.2. KISSIR Agonist Downregulates Hepatic Lipogenic Gene CIDEA

**Figure 3:**
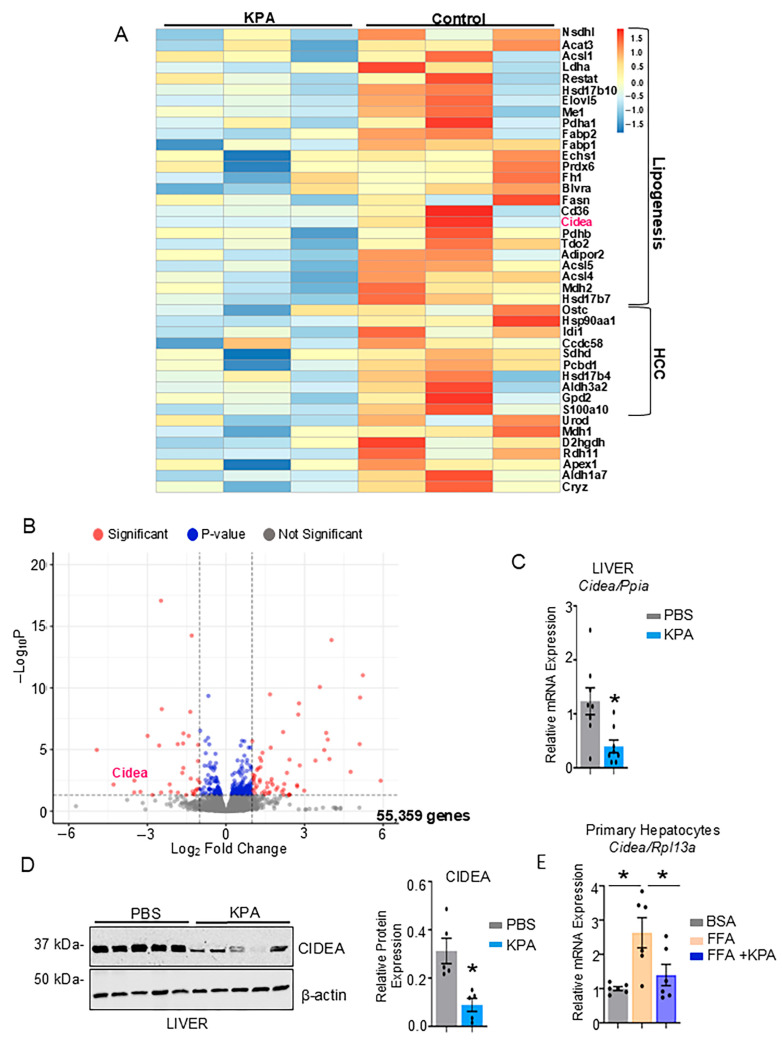
KPA treatment decreases hepatic CIDEA in steatotic livers. (**A**,**B**) Changes in gene expression in steatotic livers from DIAMOND mice on WDSW for 12 weeks as determined by bulk RNA-seq (N = 3/group). Data presented as (**A**) heatmap and (**B**) volcano plot (*p*_adj_ < 0.1). Transcriptomic analysis of KPA-treated DIAMOND mice livers identifies significant downregulation of *Cidea*. Expression of *Cidea* in steatotic livers determined by (**C**) RT-qPCR and (**D**) Western blot; the representative blot shows the expression of hepatic CIDEA in DIAMOND livers treated with KPA or PBS (vehicle); (N = 5–7/group). (**E**) Expression of hepatic *Cidea* determined by RT-qPCR in primary mouse hepatocytes treated with BSA, FFA (150 μM oleic acid and 150 μM palmitic acid) conjugated with 2% BSA in the presence or absence of 3 nM KPA (N = 6/group). Results expressed as mean ± S.E.M. Student’s unpaired *t*-test, * *p* < 0.05 versus respective controls.

To elucidate the mechanism by which KPA alleviates steatosis, bulk RNA-seq was conducted to detect changes in hepatic gene expression in KPA-treated DIAMOND mice. As observed in the heat map, several genes that regulate lipogenesis and are implicated in the development of steatosis were significantly downregulated in the KPA-treated livers (Figure 3A). These include fatty acid binding proteins (*Fabp1*, *Fabp2*) [23], fatty acid transporter (*Cd36*) [24] and fatty acid elongase (*Elovl5*) which regulates fatty acid elongation [25,26]. Gene enrichment analysis revealed that fatty acid metabolism was one of the most downregulated pathways in the KPA-treated livers (Supplemental Figure S1). Interestingly, KPA-treated livers displayed an upregulation of interferon gamma-related pathways, that has well-established antifibrogenic effects and anti-tumor effects in hepatocellular carcinoma (HCC) [27]. In fact, genes that regulate fibrosis and HCC were also significantly downregulated in the KPA-treated livers (Figure 3A); these include as calcium-binding protein A10 (*S100a10*) [28], succinate dehydrogenase subunit (*Sdhd*) [29] and peroxisome-localized enzyme *Idi1* [30] (Figure 3A).

One of the most downregulated genes was cell death-inducing DNA fragmentation factor alpha-like effector a (*Cidea)* (Figure 3B). *Cidea* promotes lipid droplet formation and is elevated in the livers of patients with steatotic disease [31,32,33]. To verify this finding further, livers from KPA-treated mice were analyzed by qPCR. Results show that *Cidea* expression was indeed significantly downregulated in KPA-treated livers (Figure 3C). Similar observations were made at the protein level by Western blot analysis (Figure 3D).

Next, to determine whether KPA downregulated CIDEA expression by directly acting on hepatocytes, lipogenesis was examined using KPA-treated primary mouse hepatocytes which express kisspeptin and KISS1R [8]. Hepatocytes isolated from DIAMOND mice on regular chow were cultured in the presence of free fatty acids (150 μM palmitic acid and 150 μM oleic acid) conjugated with BSA and treated with KPA (3 nM). This dose of KPA has been shown to decrease triglyceride accumulation in primary hepatocytes [8]. FFA treatment significantly induced *Cidea* gene expression and this was substantially reduced upon KPA treatment (Figure 3E). Thus, one mechanism by which KPA ameliorates hepatic steatosis is by negatively regulating CIDEA expression.

### 3.3. KISS1R Overexpression Improves Hepatic Steatosis and Downregulates CIDEA Expression

**Figure 4:**
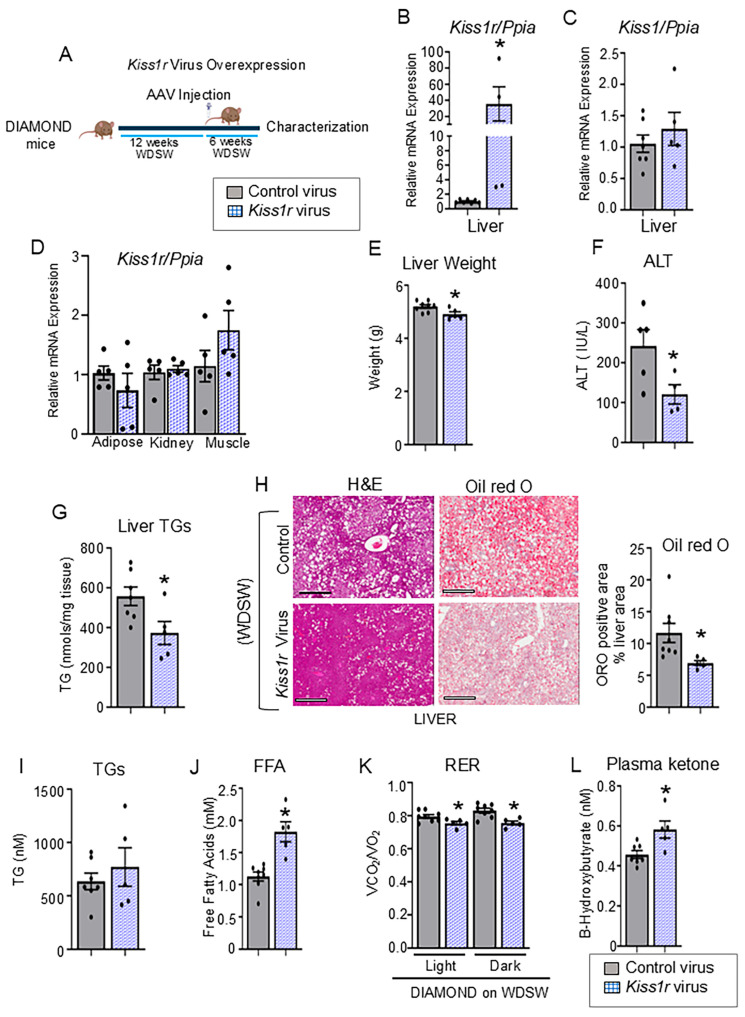
Hepatic *Kiss1r* overexpression protects against hepatic steatosis. (**A**) Schematic of timeline showing when DIAMOND mice on WDSW were injected with AAV8-TBG-m-*Kiss1r* or AAV8-TBG-eGFP control viruses (N = 5–7/group). Six weeks after viral injections, mice were euthanized, and the following measurements were taken. Gene expression of (**B**) *Kiss1r* and (**C**) *Kiss1* by RT-qPCR. Gene expression of (**D**) *Kiss1r* in other organs by RT-qPCR. Endpoint measurements: (**E**) liver weight, (**F**) serum ALT levels, (**G**) liver triglycerides. (**H**) Representative H&E (left) ORO stained (right) liver sections, and quantification of ORO. Scale bars: 400 μm. (**I**) Serum triglycerides (TGs) and (**J**) FFA. (**K**) Metabolic chambers (CLAMS) analysis of respiratory exchange ratio (RER). (**L**) Serum ketone levels. Results expressed as mean ± S.E.M. Student’s unpaired *t*-test, * *p* < 0.05 versus respective controls.

Next, we tested whether increased expression of hepatic *Kiss1r* is sufficient to improve steatosis. KISS1R expression was increased by injecting intravenously DIAMOND mice on WDSW randomly with AAV8-TBG-m-*Kiss1r* or AAV8-TBG-eGFP control viruses (Figure 4A). *Kiss1r* expression was increased in the liver, in contrast to other metabolic organs (e.g., adipose, muscle, and kidney) (Figure 4B,D). No change was observed in the hepatic *Kiss1* expression between the two groups (Figure 4C).

Importantly, *Kiss1r* overexpressing mice on WDSW exhibited lower liver weight, serum ALT levels, and liver triglycerides, and displayed significantly less hepatic steatosis (Figure 4E–H). No significant differences were observed in body weight, food intake (Supplemental Figure S2A,B) or serum triglyceride levels between the two groups (Figure 4I). Serum free fatty acids were significantly increased in *Kiss1r* overexpressing mice (Figure 4J), suggestive of decreased utilization of circulating fats, or increased lipolysis of fats in adipose tissue; the latter would suggest a decrease in white adipose tissue mass. We observed a trend towards a decrease in the weight of epididymal white adipose tissue (Supplemental Figure S2C); possibly longer duration of hepatic *Kiss1r* overexpression is required to see a significant effect on adipose tissue. No difference in muscle mass was observed between the two groups (Supplemental Figure S2D). Respiratory exchange ratio (RER) was significantly decreased in the *Kiss1r* overexpressing mice during the light and dark phases compared to controls, suggesting that *Kiss1r* overexpression increased fat utilization (Figure 4K). Consistent with these findings, hepatic β-oxidation of fats was assessed by measuring circulating ketone bodies, and results showed a significant increase in ketone levels in the *Kiss1r* overexpressing mice (Figure 4L). No significant differences were found in energy expenditure or in locomotion (Supplemental Figure S2E,F).

**Figure 5:**
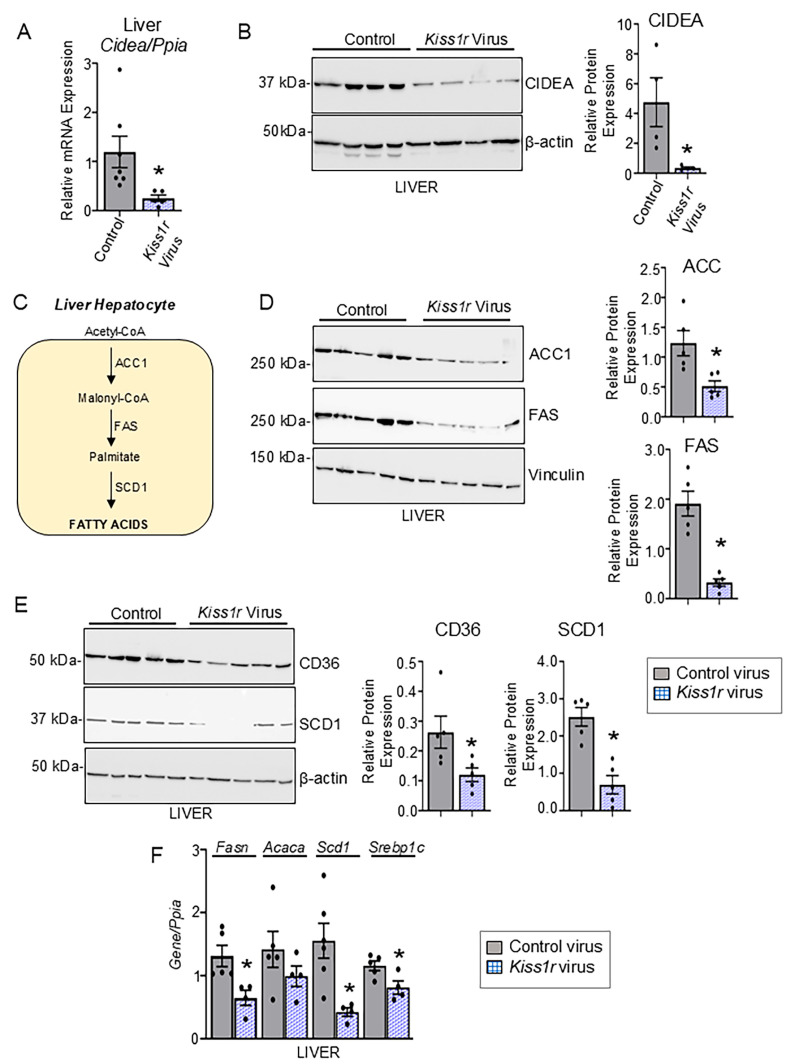
Hepatic *Kiss1r* overexpression decreases markers of de novo lipogenesis. DIAMOND mice on WDSW were injected with AAV8-TBG-m-*Kiss1r* or AAV8-TBG-eGFP control viruses (N = 5/group). Six weeks after viral infections, mice were euthanized, and the following were performed. Quantification of (**A**) hepatic *Cidea* mRNA levels by RT-qPCR and (**B**) hepatic CIDEA by Western blot analysis (representative blot shown). (**C**) Schematic showing key regulators of hepatic DNL pathway. (**D**,**E**) Representative Western blot showing expression of proteins promoting DNL, and densitometric analyses of blots. (**F**) Expression of genes regulating DNL quantified by RT-qPCR. Results expressed as mean ± S.E.M. Student’s unpaired *t*-test, * *p* < 0.05 versus respective controls.

To better understand the effect of *Kiss1r* overexpression on hepatic lipogenesis, we examined and found that there was a decreased expression of CIDEA mRNA and protein levels in *Kiss1r* overexpressing mice livers (Figure 5A,B). Protein expression of key regulators of DNL, such as acetyl-CoA carboxylase (ACC1), which catalyzes the rate-limiting step of DNL by converting acetyl-CoA to malonyl-CoA (Figure 5C), was also significantly reduced in *Kiss1r* overexpressing livers (Figure 5D), and we observed a trend toward decreased *Acaca* mRNA level (encodes ACC1) (Figure 5F). The gene (*Fasn*) and protein expression of fatty acid synthase (FAS), which catalyzes de novo synthesis of long-chain fatty acids, was also significantly reduced in the *Kiss1r* overexpressing mice livers (Figure 5D,F). Stearoyl-CoA desaturase 1 (SCD1) is the rate-limiting enzyme of biosynthesis of monounsaturated fatty acids, which serve as substrates for de novo lipogenesis, increasing the synthesis of triglycerides in the liver. SCD1 expression was significantly downregulated at the gene and protein level, in *Kiss1r* overexpressing mice livers (Figure 5E,F). Additionally, it was observed that the livers of *Kiss1r* overexpressing mice had significantly reduced expression of the fatty acid translocase, CD36 (Figure 5E). CD36 uptakes FFA and thereby promotes hepatic steatosis [34]. These data demonstrate that KISS1R overexpression can critically reverse hepatic lipogenesis.

### 3.4. KISS1R Signaling Inhibits the Expression of the Active Form SREBP-1c

**Figure 6:**
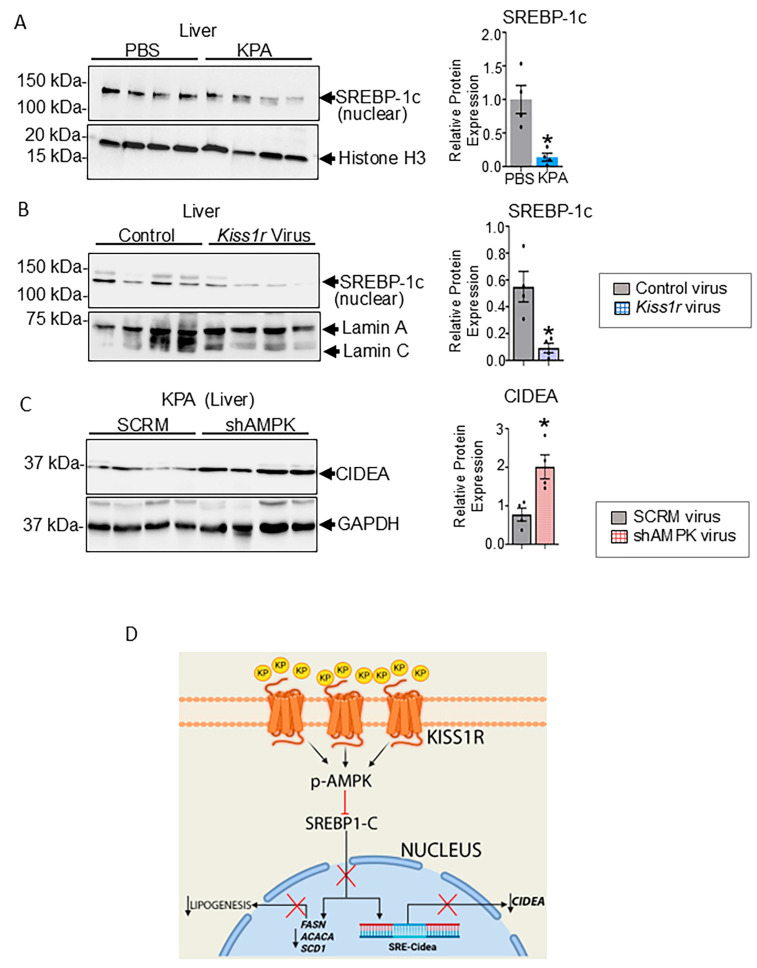
KISS1R signaling reduces SREBP-1c protein levels. Representative Western blots showing the expression of hepatic SREBP-1c in nuclear lysates from DIAMOND mice on WDSW (for 12 weeks), (**A**) treated with KPA or PBS (vehicle) for 6 weeks (N = 4/group) and (**B**) in mice injected with AAV8-TBG-m-*Kiss1r* or AAV8-TBG-eGFP control viruses (N = 4/group). (**C**) Representative Western blot showing the expression of CIDEA in liver lysates from C57BL/6J mice fed HFD for 4 weeks, then injected with either AAV8-U6-M-PRKAA2 shRNA (shAMPK) or AAV8-U6-M-SCRM shRNA (SCRM); mice were kept on HFD for 3 weeks before KPA treatment was carried out for 6 weeks, in addition to maintaining HFD (N = 4/group). (**D**) Schematic showing proposed signaling pathways by which KISS1R signaling suppresses hepatic DNL: enhanced activation of KISS1R activates AMPK, thereby reducing SREBP-1c levels, and attenuating the expression of genes regulating DNL, including *Cidea*. Results expressed as mean ± S.E.M. Student’s unpaired *t*-test, * *p* < 0.05 versus respective controls.

The transcription factor sterol regulatory element binding protein 1c (SREBP-1c) is a critical regulator of hepatic DNL [35], by transcriptionally upregulating lipogenic genes such as *Acaca*, *Fasn*, and *Scd*1 [36]. SREBP-1c can upregulate the CIDEA expression by binding sterol-regulatory elements (SRE) in the CIDEA promoter [37,38,39]. We therefore examined the protein expression of the active (nuclear) form of SREBP-1c in KPA-treated DIAMOND mice livers and found that KPA significantly decreased the expression of hepatic SREBP-1c in nuclear extracts (Figure 6A). Consistent with this finding, nuclear SREBP-1c protein levels were also significantly blunted in livers from *Kiss1r* overexpressing mice (Figure 6B). Furthermore, decreased hepatic *Srebp-1c* gene expression was also observed in *Kiss1r* overexpressing mice (Figure 5F). No significant differences were observed in cytosolic SREBP-1c levels in KPA-treated DIAMOND mice livers or in hepatic *Kiss1r* overexpressing mice livers (Supplemental Figure S3A and Figure S3B, respectively). Since KPA activates AMPK to thereby decrease steatosis [8], we evaluated the effect of a liver-specific AMPK deletion (shAMPK) on CIDEA expression; we have previously characterized the effect of KPA-treatment on shAMPK mice [8]. We observed that CIDEA expression was significantly higher in KPA-treated shAMPK-mice (Figure 6C), implicating a role for AMPK signaling downstream of KPA to downregulate CIDEA via SREBP-1c to thereby inhibit hepatic lipogenesis (Figure 6D).

## 4. Discussion

In this study, we present the first evidence using a metabolic tracer that enhanced kisspeptin signaling suppresses hepatic de novo lipogenesis (DNL), a metabolic pathway responsible for fatty acid synthesis that contributes to intrahepatic lipid accumulation and triglyceride production. We identify the lipid droplet-associated protein CIDEA as a new target for hepatic kisspeptin/KISS1R signaling. Mechanistically, we demonstrate for the first time that KISS1R signaling negatively regulates the protein expression of the mature (nuclear) form of master lipogenic transcription factor, SREBP-1c, that induces the expression of lipogenic proteins like CIDEA, ACC1, and SCD1 to promote the fatty acid and triglyceride synthesis in the liver, thereby increasing DNL (Figure 6D).

DNL increases with the severity of MASLD in patients [18], and is a clinical therapeutic target of many drugs, including resmetirom, the first FDA-approved drug to treat this disease, which has shown disease resolution in 30% of patients [40,41,42]. We have previously shown that hepatic KISS1R signaling suppresses the development of MASLD by increasing the b-oxidation of fat via activation of hepatic AMPK thereby attenuating hepatic lipogenesis [8]. However, whether kisspeptin can directly decrease hepatic DNL, a subset of lipogenesis that measures fatty acid synthesis from excess carbohydrates, was not known [43]. Hydrogen isotope tracers of water (^2^H_2_O) have been extensively used as metabolic tracers to study DNL in vivo; besides being safe and rapidly equilibrating after ingestion, the enrichment diminishes very slowly following initial administration [44]. Using a mouse model of MASLD, we found that enhancing KISS1R activation by administering a long-acting kisspeptin analog, KPA [45], decreases the synthesis of numerous lipid species shown to be linked to obesity, type 2 diabetes, and steatosis, in addition to HCC [21,22]. The synthesis of palmitate (C16:0) was visibly reduced, although significance was not achieved; this could be due to the length of the KPA treatment and the need for a longer treatment duration. Additionally, many fatty acids such as palmitic acid, oleic acid (C18:1), stearic acid (C18:0), and myristic acid (C14:0) are present in the diet used to induce MASLD in DIAMOND mice [TD.88137; Envigo RMS, Inc. Indianapolis, IN, USA]. Thus, it is possible that ingestion of these unlabeled fatty acids, followed by their incorporation into hepatic triglycerides, mitigated the observed potential differences in deuterium enrichment.

CIDEA regulates lipid droplet fusion, growth, and storage in human and mouse adipocytes [46]. In white adipose tissues from patients with obesity and insulin resistance, CIDEA expression is linked to high triglyceride accumulation [32,47]. Additionally, in patients exhibiting obesity and hepatic steatosis, CIDEA expression, which is induced by saturated fatty acids, is elevated in hepatocytes [33,48]. In parallel, studies in mice using diet-induced obesity models (e.g., using high-fat diet-fed mice or the ob/ob mice) report an increase in hepatic CIDEA in steatotic livers, and conversely, *Cidea* knockdown decreased hepatic steatosis [33,49,50,51].

DNL is promoted via hyperglycemia and hyperinsulinemia. Insulin activates SREBP-1c, which plays a vital role in promoting the conversion of glucose to lipid [52]. SREBP-1c transcriptionally regulates the expression of *Cidea*, and sterol-regulatory elements (SREs) are localized to the *Cidea* promoter [33,39]. Enhancing KISS1R signaling pharmacologically and genetically by the overexpression of hepatic *Kiss1r* results in downregulation of CIDEA and SREBP-1c activity, providing a mechanistic mediator of the beneficial role of kisspeptin/KISS1R signaling.

SREBP-1c expression and activity are suppressed by the master energy sensor AMPK, leading to a reduction in hepatic steatosis [53,54]. We have previously shown that KPA activates AMPK directly, in isolated primary hepatocytes, and KPA-treated HFD-fed mice livers [8]. Liver-specific deletion of AMPK negated the effects of KPA on hepatic steatosis and progression of MASLD [8]. Thus, activation of hepatic AMPK downstream of KISS1R can alleviate DNL by decreasing SREBP-1c levels, thereby decreasing CIDEA and lipogenic genes involved in hepatic steatosis (Figure 6D).

Besides regulating DNL, SREBP-1c expression, activation, and stability have been implicated in HCC tumor proliferation and invasion, leading to tumor growth and metastasis [55]. Elevated SREBP-1c levels found in patients with HCC tumors correlated with poor survival [56], and the SREBP-1c inhibitor betulin has been shown to have anti-tumor effects in HCC [56]. Interestingly, through the metabolic tracer study, we found that the livers of KPA-treated DIAMOND mice on WDSW exhibited a decrease in free fatty acids implicated in HCC (e.g., C20:1, C22:1, C24:3 and C24:4) [22]. Additionally, by RNA-seq, several genes implicated in HCC were downregulated in KPA-treated DIAMOND livers. These include 17β-hydroxysteroid dehydrogenase 4 *Hsd17b*4 [57], heat shock protein *Hsp90aa* [58], and aldehyde dehydrogenase *Ald3a* [59]. Taken together, this suggests that KPA-treatment, in addition to reducing DNL and improving steatosis, also likely inhibits the development of HCC; the latter remains to be tested. This initial work was conducted using male DIAMOND mice, since MALSD is more prevalent in males than in females [9]. Our future studies will include female mice to study kisspeptin/KISS1R signaling in MASLD. Additionally, further studies will examine the effect of knocking out *Srebp-1c* or *Cidea* in mice treated with kisspeptin to advance our hypothesis. Since SREBP-1c has been implicated to play a role in HCC, a longer treatment of kisspeptin will reveal whether kisspeptin/KISS1R signaling can downregulate SREBP-1c levels in advanced disease and HCC.

In summary, this study reveals that KISS1R signaling inhibits hepatic DNL, reducing the production of FFA linked to obesity, steatosis, and HCC. Mechanistically, this occurs via a reduction in the expression of hepatic CIDEA levels by decreasing the expression of SREBP-1c. Our data suggest that future studies involving the administration of kisspeptin and metabolic tracers such as ^2^H_2_O-enriched water in obese patients with steatosis are warranted to determine whether KPA also regulates DNL in humans. We have recently shown that the activation of KISS1R can decrease fibrosis in patient livers, via the inactivation of transforming growth factor (TGF)b signaling in hepatic stellate cells, the main drivers of hepatic fibrosis [16]. Taken together, this suggests that kisspeptin peptides have a multifaceted role in the liver and may be a viable therapeutic strategy in the prevention and treatment of MASLD.

## 5. Conclusions

In this study, we demonstrate for the first time that kisspeptin treatment in a mouse model of MASLD inhibits hepatic DNL, using a metabolic tracer. Mechanistically, we demonstrate that kisspeptin induces the downregulation of CIDEA. Importantly, we find that KISS1R signaling decreases hepatic levels of active SREBP-1c, a transcription factor that is a master upregulator of genes involved in DNL, including CIDEA.

## Data Availability

RNA-seq dataset has been deposited to GEO (Gene Expression Omnibus). It will be publicly available once this manuscript is published.

## Supplementary Materials

The following supporting information can be downloaded at: https://www.mdpi.com/article/10.3390/cells14161289/s1, Figure S1: KPA downregulates molecular pathways related to fatty acid metabolism. Figure S2: Characteristics of DIAMOND mice on WDSW overexpressing Kiss1r. Figure S3: Cytosolic expression of SREBP-1c; Table S1: Primers; Table S2: Antibodies.

## Abbreviations

The following abbreviations are used in this manuscript:

ACC1	Acetyl CoA Carboxylase
AMPK	AMP-activated protein kinase
Ald3a	Aldehyde dehydrogenase
AUC	Area under the curve
BSA	Bovine serum albumin
CD36	Cluster of differentiation (fatty acid transporter)
CIDEA	Cell death-inducing DNA fragmentation-alpha-like effector a
CLAMS	Comprehensive Laboratory Animal Monitoring System
DIAMOND	Diet-Induced Animal Model of Non-Alcoholic Liver Disease
DNL	De novo lipogenesis
HCC	Hepatocellular carcinoma
HFD	High-fat diet
^2^H_2_O	Deuterated water
Hsd17b4	17β-hydroxysteroid dehydrogenase 4
Elovl5	Elongase of very long chain fatty acids 5
FASN	Fatty acid synthase
FFA	Free fatty acids
GTT	Glucose Tolerance Test
ITT	Insulin Tolerance Test
KISS1R	Kisspeptin 1 receptor
KPA	Kisspeptin analog
LC-MS	Liquid chromatography-mass spectrometry
MASLD	Metabolic dysfunction-associated steatotic liver disease
MASH	Metabolic dysfunction-associated steatohepatitis
NAFLD	Non-alcoholic fatty liver disease
ORO	Oil Red O
RER	Respiratory ratio
SCD1	Stearoyl-CoA desaturase 1
ALT	Alanine aminotransferase
SDHD	Succinate dehydrogenase subunit
SREBP1	Sterol regulatory element-binding protein 1
SRE	Sterol-regulatory elements
S100a10	calcium-binding protein A10
TGFβ	Transforming growth factor β
TG	Triglyceride
WDSW	Western diet, sugar water
