# Supplementary material for: Kisspeptin Mitigates Hepatic De Novo Lipogenesis in Metabolic Dysfunction-Associated Steatotic Liver Disease
Source: Cells. 2025 Aug 20;14(16):1289. doi: 10.3390/cells14161289 (PMC12384258; doi:10.3390/cells14161289)
Supplement: Supplementary file 1 [file cells-14-01289-s001.zip › Izarraras et al Suppl fig legends.pdf]

SUPPLEMENTAL FIGURE LEGENDS

Supplementary Figure 1. KPA downregulates molecular pathways related to fatty acid metabolism. (A) Gene set enrichment analysis from RNA seq in steatotic livers from DIAMOND mice on WDSW for 12 weeks (N=3/group) treated with KPA or PBS (vehicle) for 6 weeks. The x-axis represents normalized enrichment scores of gene sets (green: downregulated pathways; orange: upregulated pathways). Results expressed as mean +/- S.E.M. Student's unpaired t test, *P < 0.05 versus respective controls.

Supplementary Figure 2. Characteristics of DIAMOND mice on WDSW overexpressing Kiss1r. (A) End point body weight. (B) Food intake. Weight of (C) epididymal white adipose tissue (eWAT), and (D) muscle. (E) Energy expenditure, and (F) ambulatory assessed using CLAMS. Results expressed as mean +/- S.E.M. (N= 5-7/group). Student's unpaired t test, *P < 0.05 versus respective controls.

Supplementary Figure 3. Cytosolic expression of SREBP-1c. Representative Western blots showing the expression of hepatic SREBP1-c in cytosolic lysates from DIAMOND mice on WDSW (for 12 weeks) (A) treated with KPA or PBS (vehicle) for 6 weeks and (B) in mice injected with AAV8-TBG-m-Kiss1r or AAV8-TBG-eGFP control viruses. Results expressed as mean +/- S.E.M. Student's unpaired t test, *P < 0.05 versus respective controls.
