# Supplementary material for: Kisspeptin Mitigates Hepatic De Novo Lipogenesis in Metabolic Dysfunction-Associated Steatotic Liver Disease
Source: Cells. 2025 Aug 20;14(16):1289. doi: 10.3390/cells14161289 (PMC12384258; doi:10.3390/cells14161289)

Izarraras Shah et al., BLOTS

FIGURE 3D

CIDEA expression in Diamond mice with PBS or KPA treatment

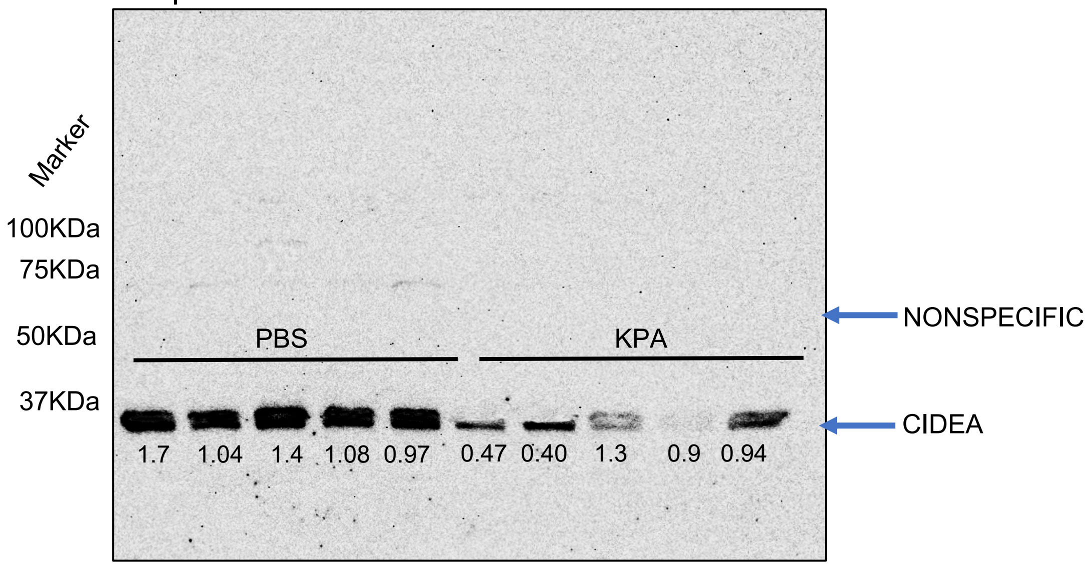

N=5 mice each group

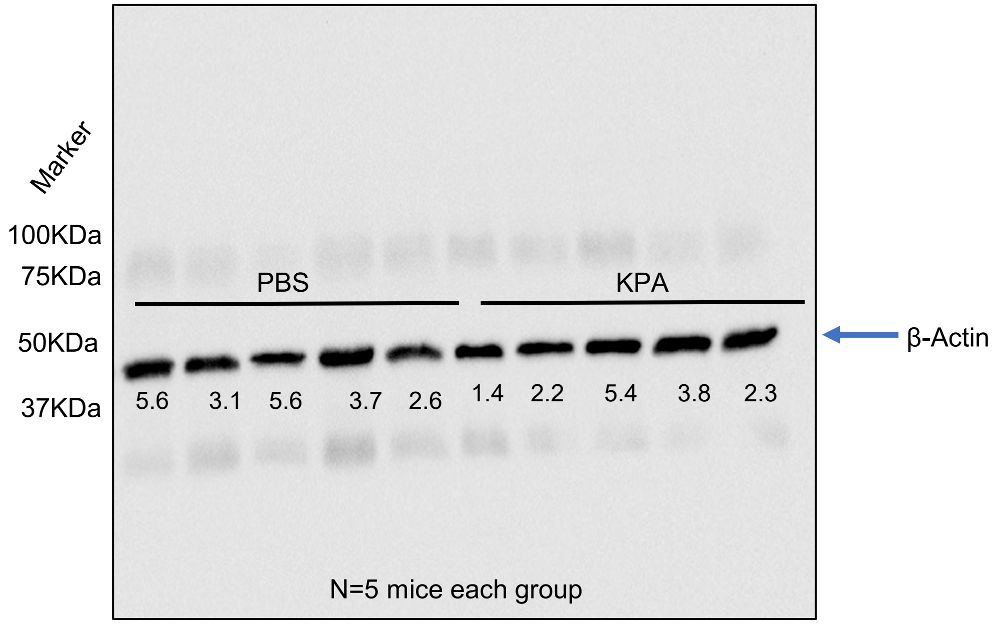

FIGURE 5B

CIDEA expression in Diamond mice with Control or *Kiss1r* overexpression

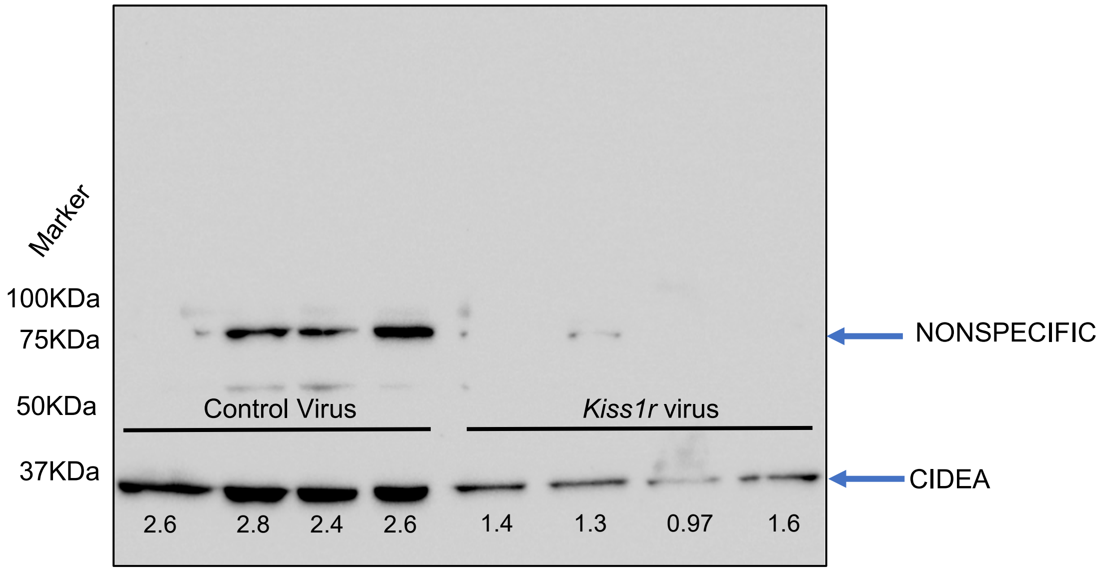

N=4 mice each group

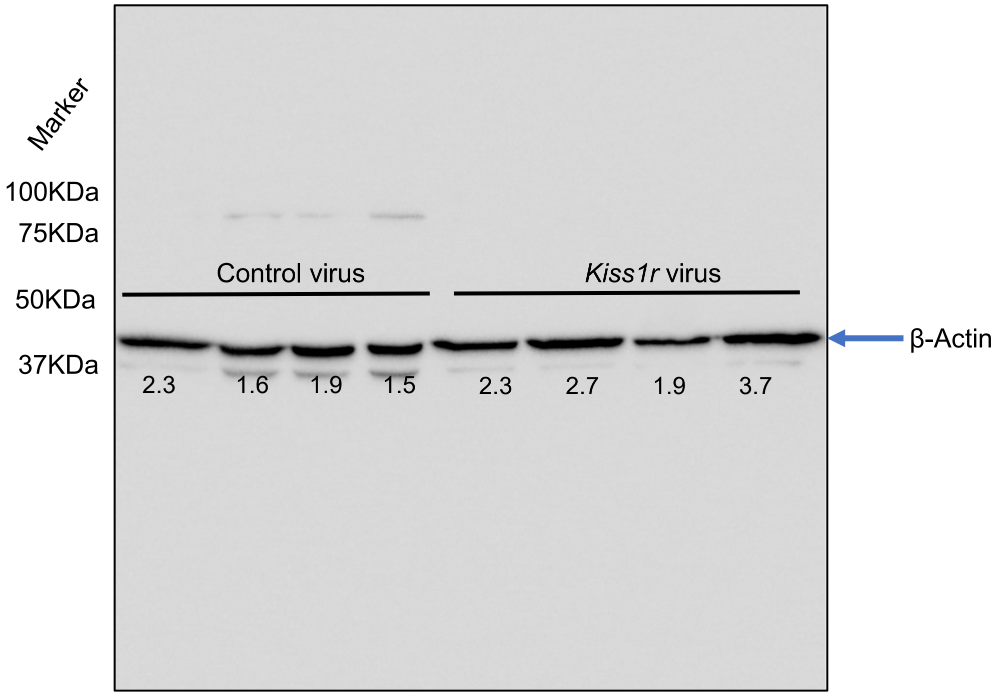

Izarraras Shah et al., BLOTS

FIGURE 5D

ACC1 expression in Diamond mice with Control or *Kiss1r* overexpression

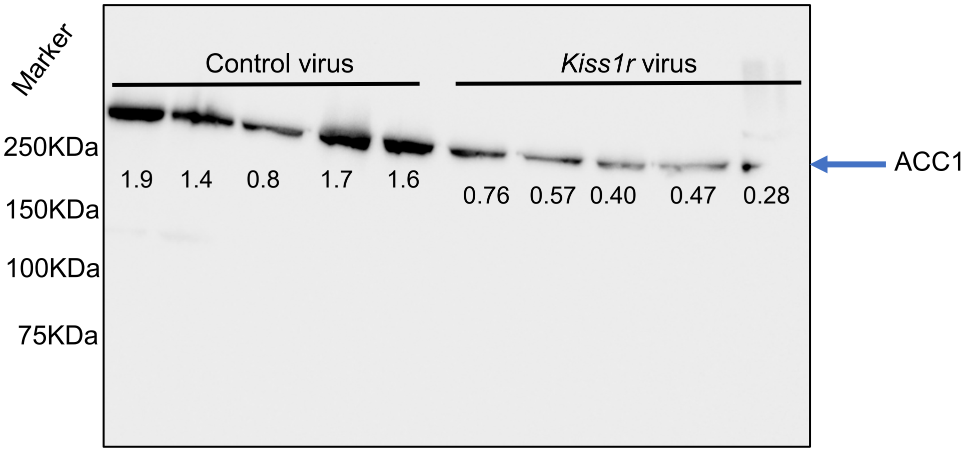

N=5 mice each group

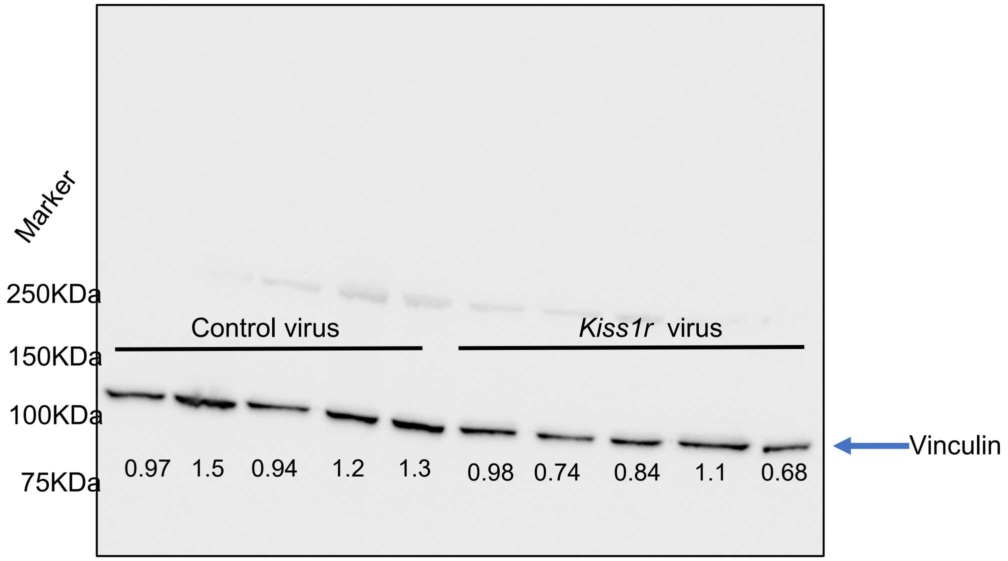

Izarraras Shah et al., BLOTS

FIGURE 5D

FAS expression in Diamond mice with Control or *Kiss1r* overexpression

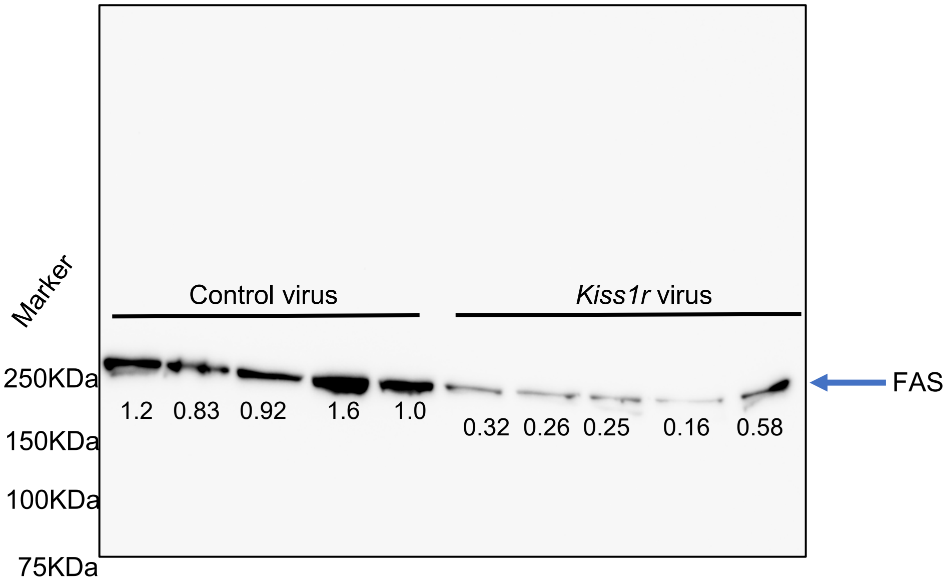

N=5 mice each group

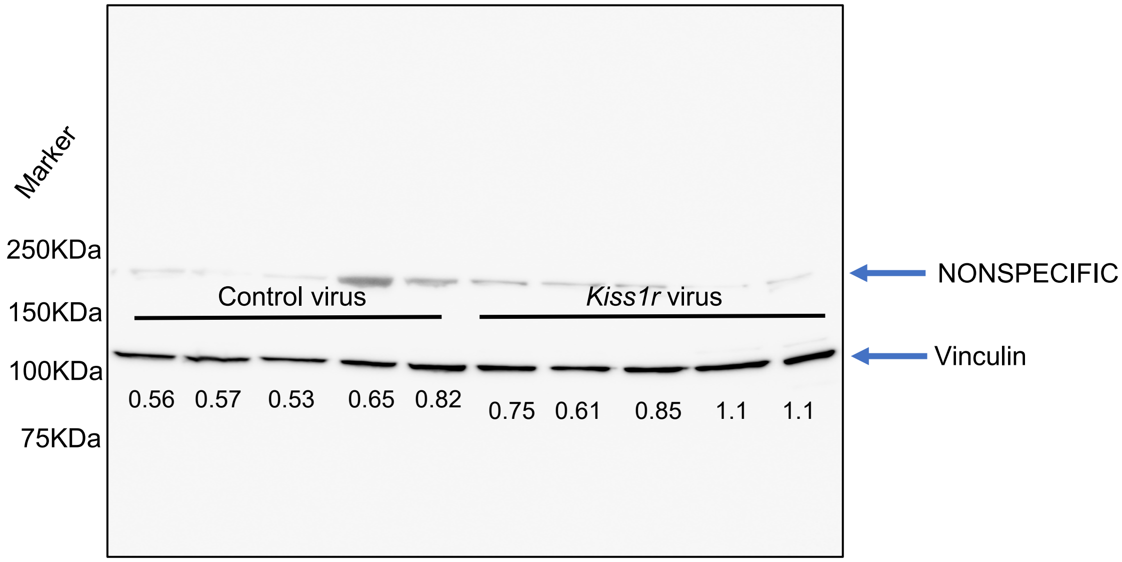

Izarraras Shah et al., BLOTS

FIGURE 5E

CD36 expression in Diamond mice with Control or *Kiss1r* overexpression

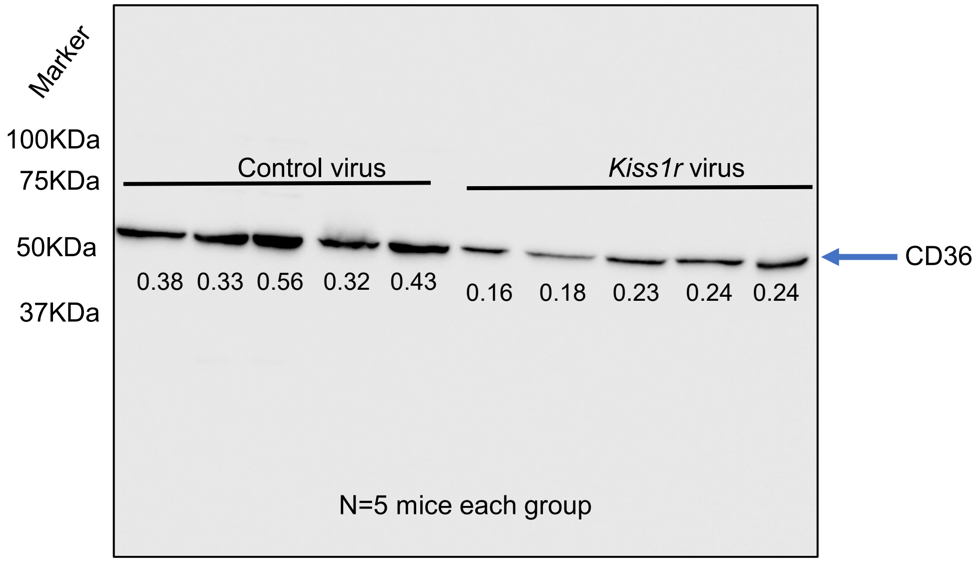

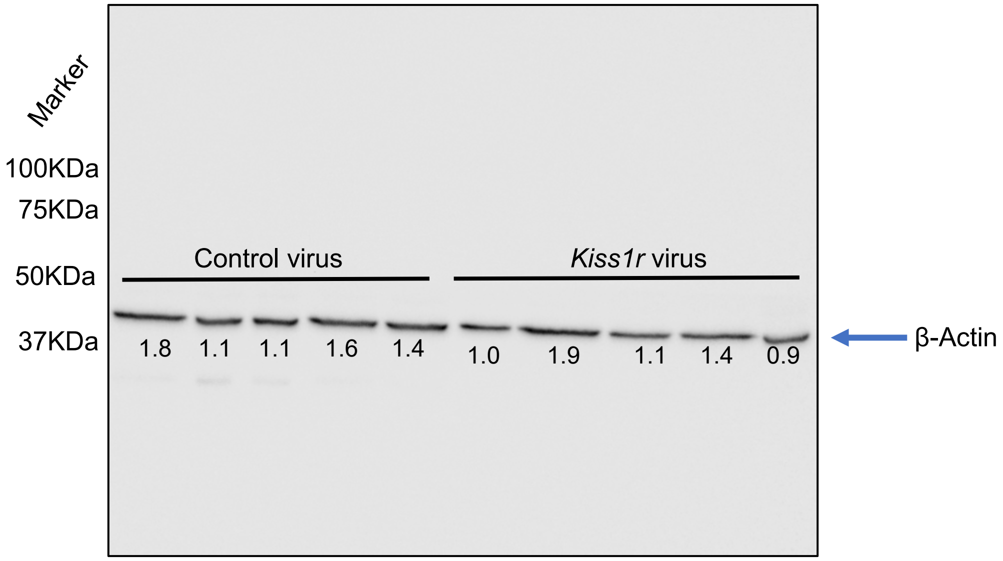

Izarraras Shah et al., BLOTS

FIGURE 5E

SCD1 expression in Diamond mice with Control or *Kiss1r* overexpression

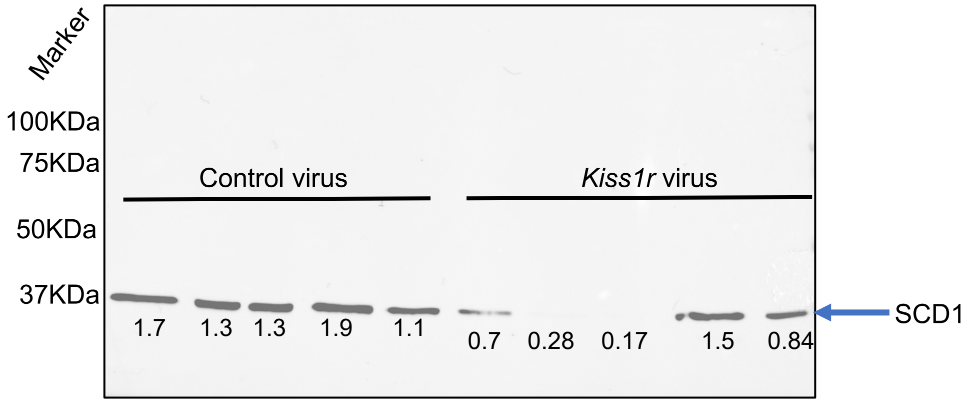

N=5 mice each group

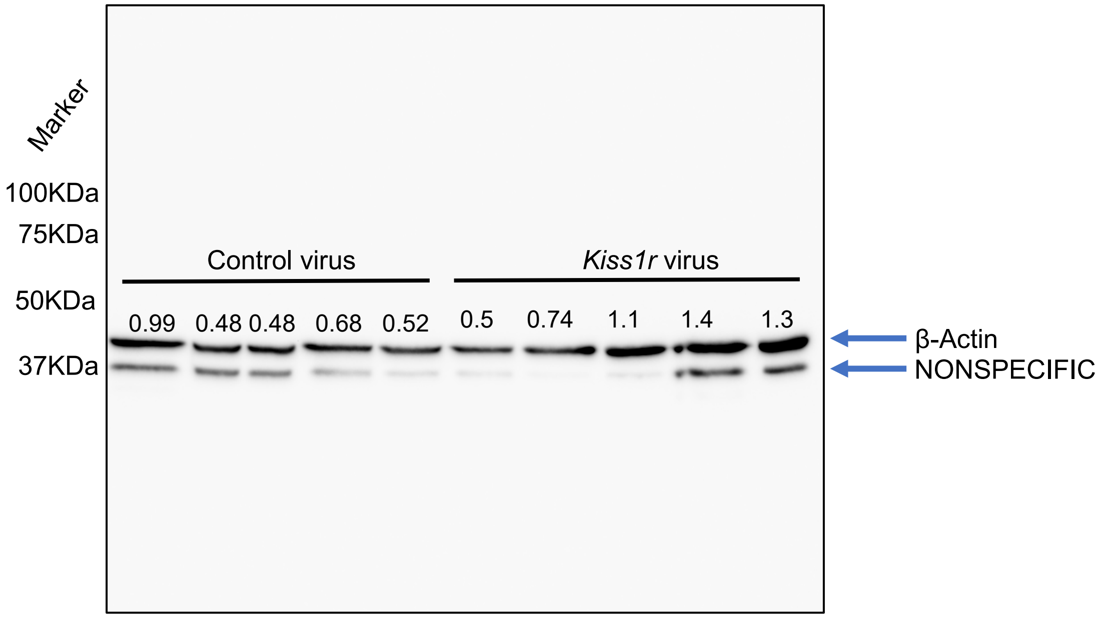

Izarraras Shah et al., BLOTS

FIGURE 6A

SREBP-1c (nuclear) expression in Diamond mice with PBS or KPA

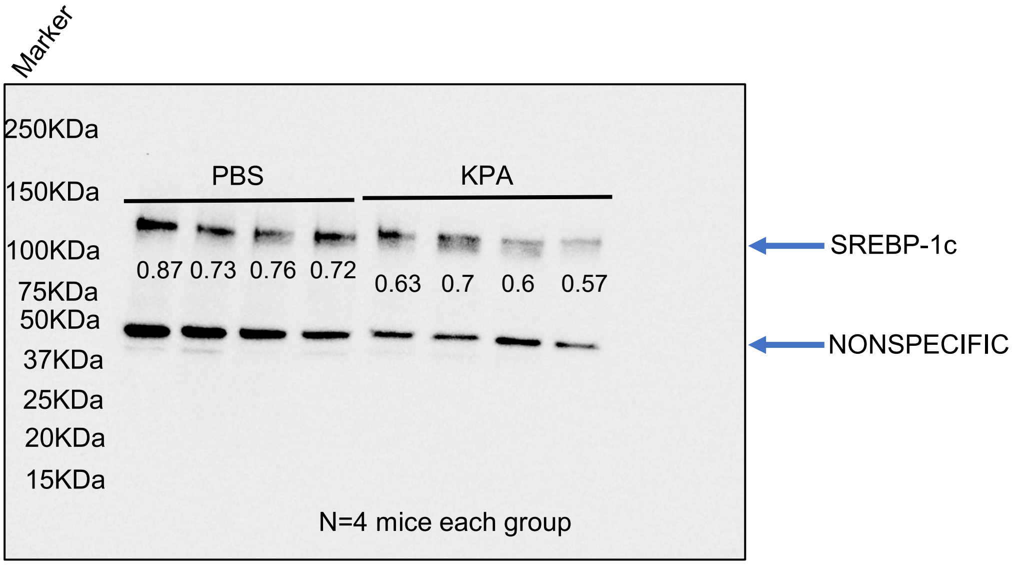

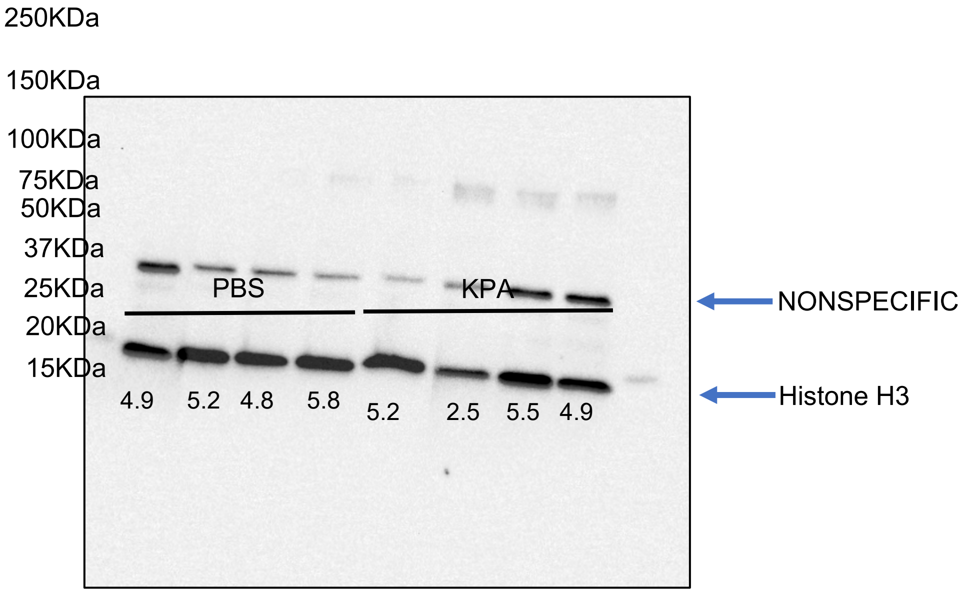

Izarraras Shah et al., BLOTS FIGURE 6B

SREBP-1c (Nuclear) expression in Diamond mice with Control or *Kiss1r* overexpression

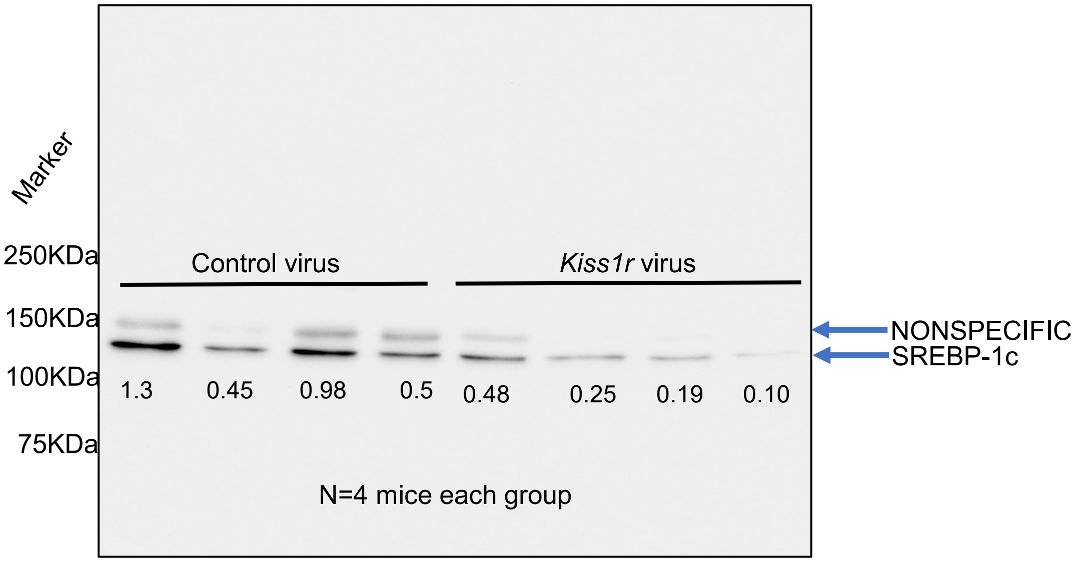

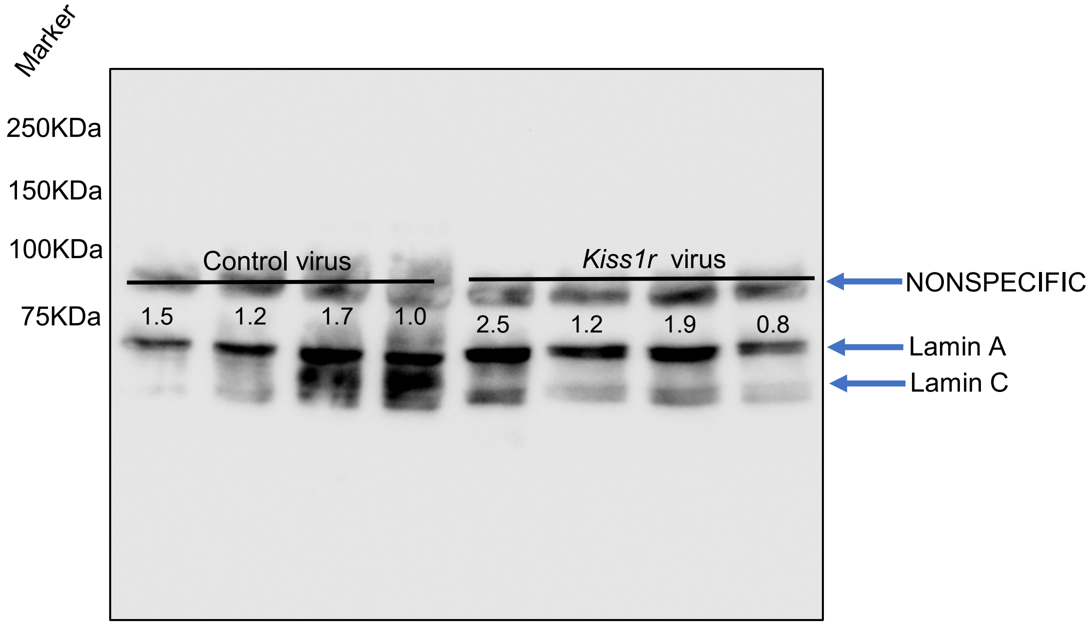

FIGURE 6C

CIDEA (Cytosol) expression in C57BL/6J mice injected with shAMPK or SCRM (control) virus and treated with KPA

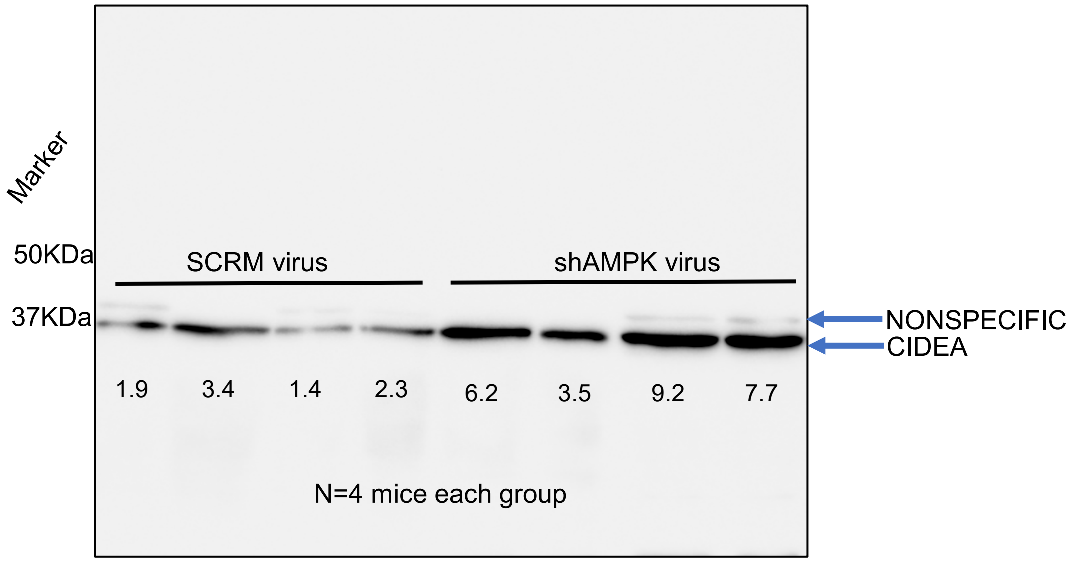

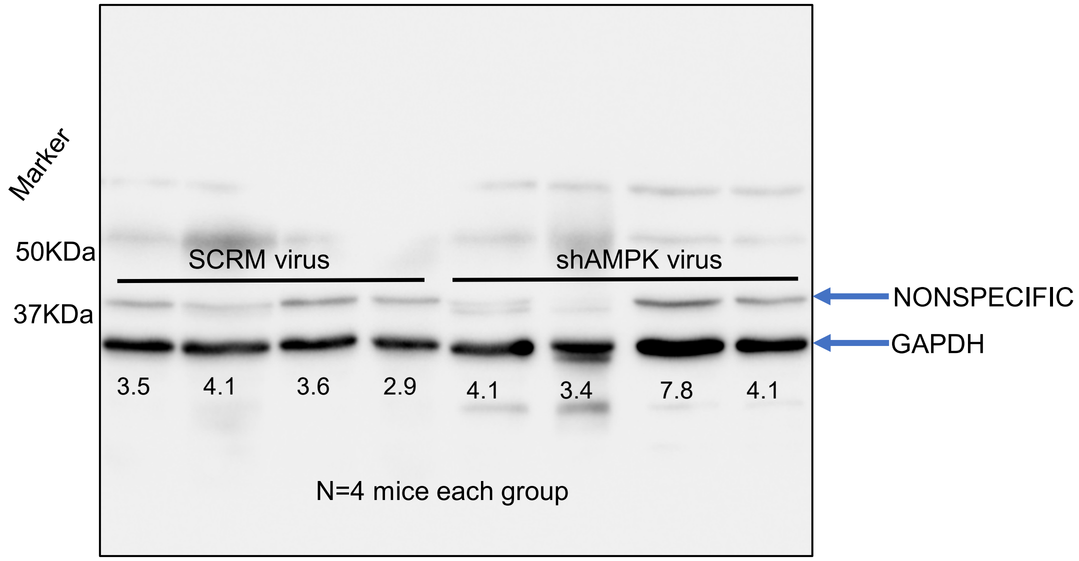

Izarraras Shah et al., BLOTS

Supplementary FIGURE 3A

SREBP-1c (cytosol) expression in Diamond mice with PBS or KPA

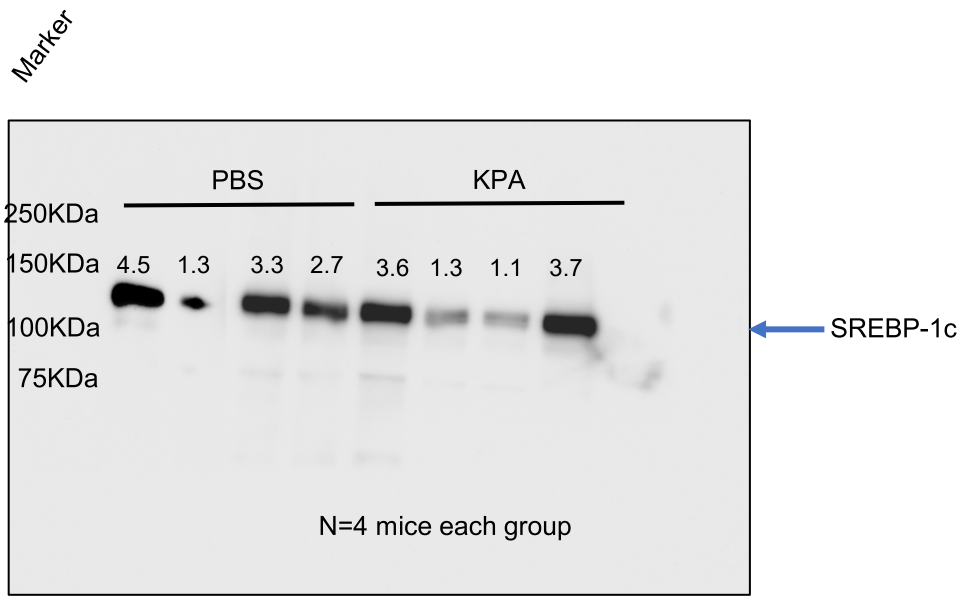

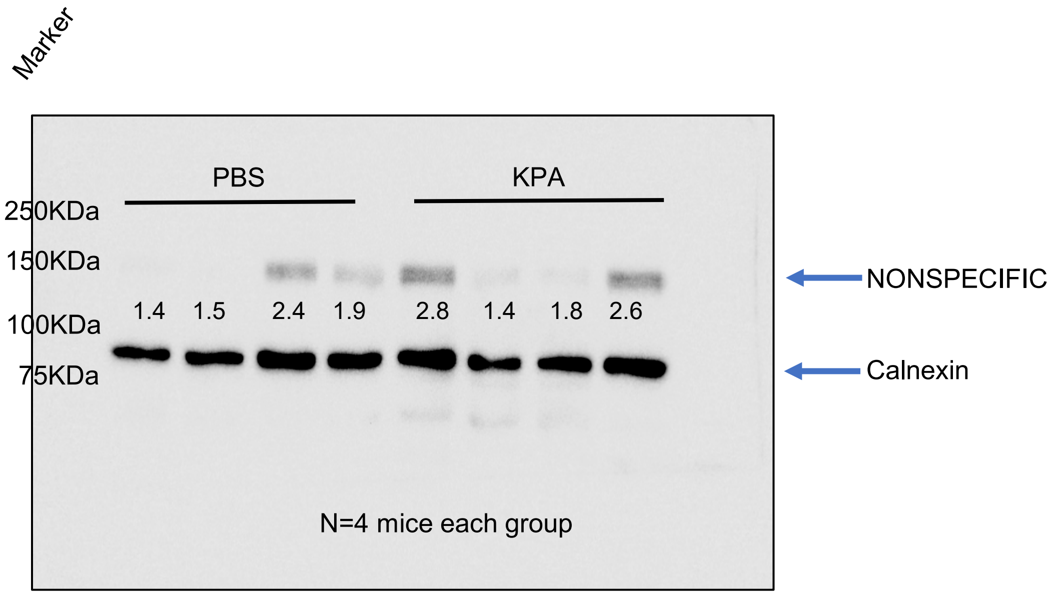

Izarraras Shah et al., BLOTS

SUPPLEMENTARY FIGURE 3B

SREBP-1c (Cytosol) expression in Diamond mice with Control or *Kiss1r* overexpression

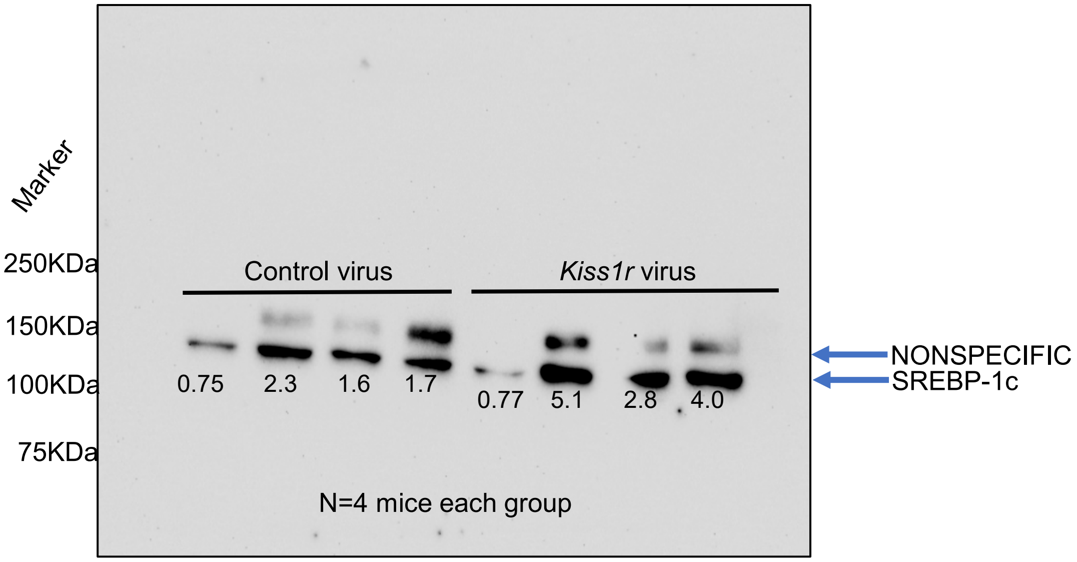

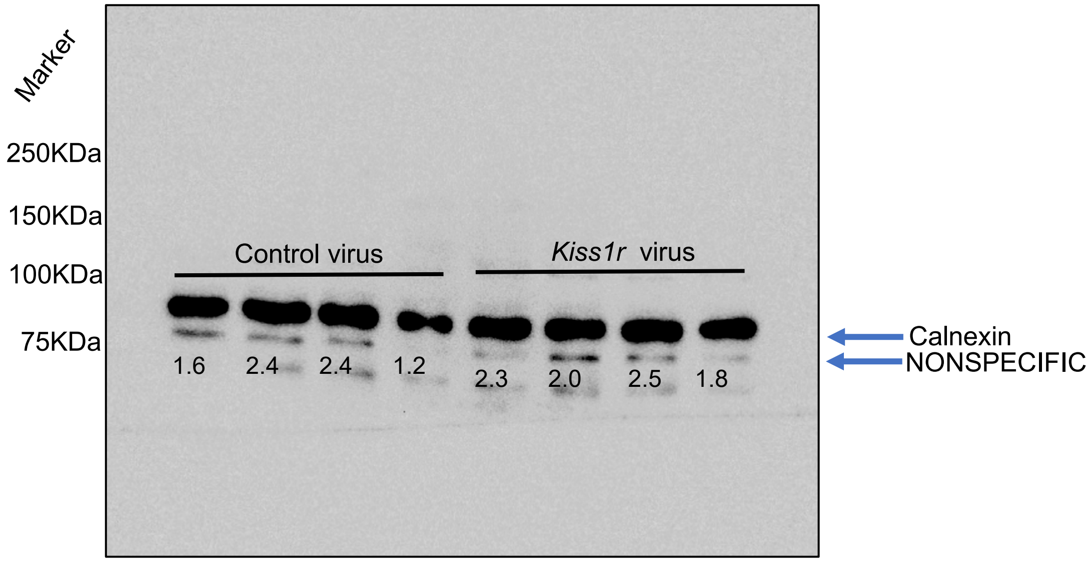

Supplement: Supplementary file 1 [file cells-14-01289-s001.zip › Izarraras Shah full blots (REVISED).pdf]
