# Supplementary material for: Kisspeptin Mitigates Hepatic De Novo Lipogenesis in Metabolic Dysfunction-Associated Steatotic Liver Disease
Source: Cells. 2025 Aug 20;14(16):1289. doi: 10.3390/cells14161289 (PMC12384258; doi:10.3390/cells14161289)
Supplement: Supplementary file 1 [file cells-14-01289-s001.zip › Supplemental Table 1.pdf]

Supplemental Table 1:Primers

Name	Forward	Reverse
Kiss1	AGCTGCTGCTTCTCCTCTGT	GCATACCGCGATTCCTTTT
Kiss1r	CTGCCACAGACGTCACTTTC	ACATACCAGCGGTCCACACT
Cidea	AAGCTTCAAGGCCGTGTT	CTGTAGCTGTGCCCTGGTTA
Scd1	TCTTCCTTATCATTGCCAACACCA	GCGTTGAGCACCAGAGTGTATCG
Srebp1c	CACTTCTGGAGACATCGCAAAC	ATGGTAGACAACAGCCGCATC
Fasn	GGAGGTGGTGATAGCCGGTAT	TGGGTAATCCATAGAGCCCAG
Acaca	ATGGGCGGAATGGTCTCTTTC	TGGGGACCTTGTCTTCATCAT
Ppia	CAGACGCCACTGTCGCTTT	TGTCTTTGGAACTTTGTCTGCAA
Rpl13a	GCTGCTCTCAAGGTTGTTCG	CCTTTTCCTTCCGTTTCTCC
