# Supplementary material for: Kisspeptin Mitigates Hepatic De Novo Lipogenesis in Metabolic Dysfunction-Associated Steatotic Liver Disease
Source: Cells. 2025 Aug 20;14(16):1289. doi: 10.3390/cells14161289 (PMC12384258; doi:10.3390/cells14161289)
Supplement: Supplementary file 1 [file cells-14-01289-s001.zip › Supplemental Table 2.pdf]

Supplemental Table 2:Antibodies

Name	Source	Company	Catalog #	Dilution
Cidea	Rabbit	Abcam	Ab8402	1:500 5%Milk
SCD1	Rabbit	CST	2794s	1:1000 5%BSA
FAS	Rabbit	CST	3189s	1:1000 5%BSA
ACC	Rabbit	CST	4190s	1:1000 5%BSA
CD36	Rabbit	Abcam	Ab133625	1:1000 5%BSA
SREBP-1c	Rabbit	Abcam	Ab28481	1:1000 5%BSA
B-Actin	Rabbit	CST	4970s	1:1000 5%Milk
Lamin	Mouse	CST	4777s	1:2000 5%Milk
Histone H3	Rabbit	CST	4499T	1:2000 5%Milk
Vinculin	Rabbit	CST	13901s	1:1000 5%BSA
HRP-Conjugated	Rabbit	CST	7074s	1:1000 5%Milk
HRP-Conjugated	Mouse	CST	7076s	1:1000 5%Milk
