# Supplementary figures and images for: Kisspeptin Mitigates Hepatic De Novo Lipogenesis in Metabolic Dysfunction-Associated Steatotic Liver Disease
Source: Cells. 2025 Aug 20;14(16):1289. doi: 10.3390/cells14161289 (PMC12384258; doi:10.3390/cells14161289)

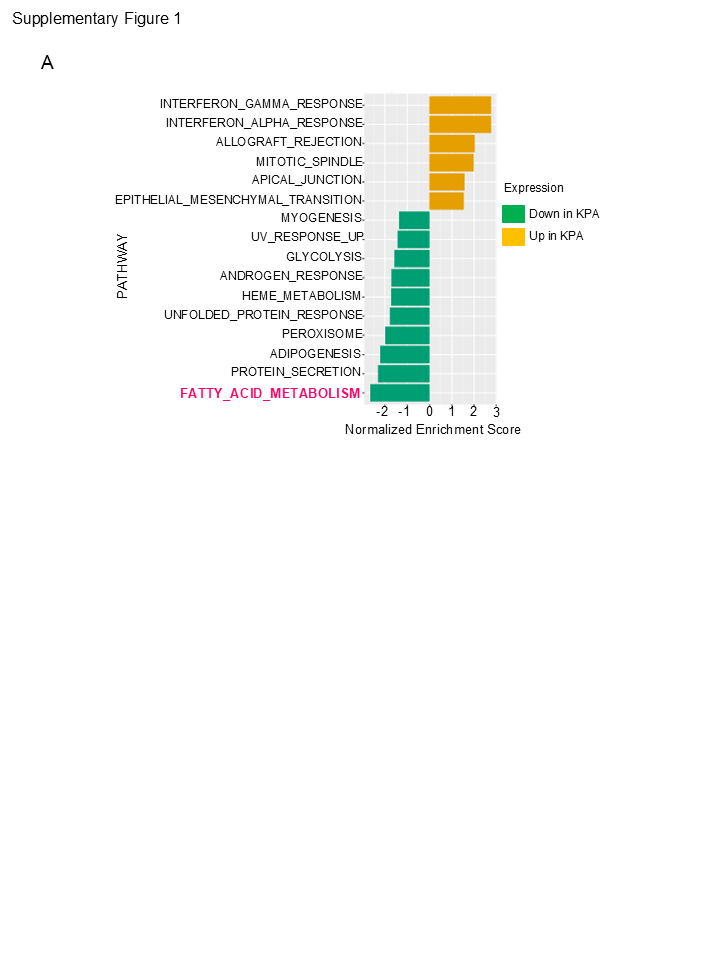

Supplement: Supplementary file 1 [file cells-14-01289-s001.zip › SUPPLEMENTAL FIGURE 1.tif]

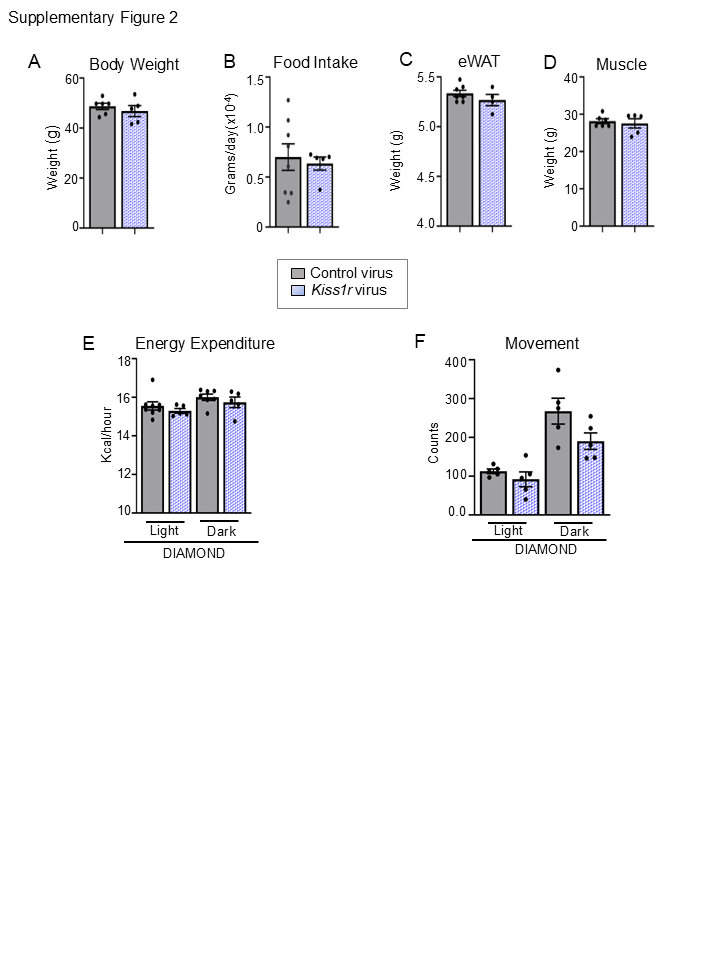

Supplement: Supplementary file 1 [file cells-14-01289-s001.zip › SUPPLEMENTAL FIGURE 2.tif]

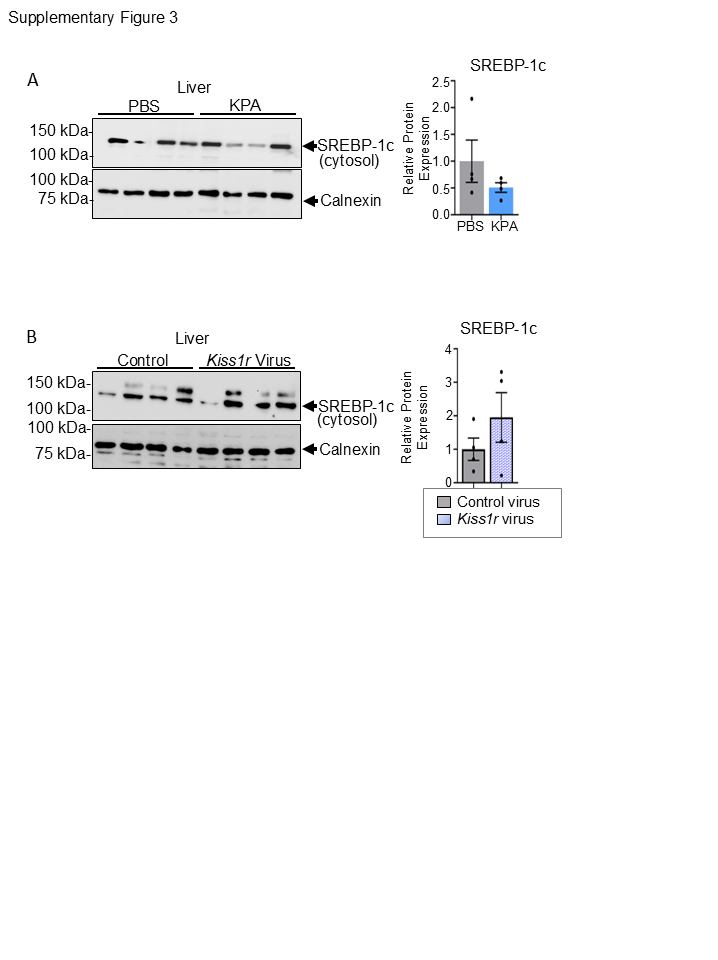

Supplement: Supplementary file 1 [file cells-14-01289-s001.zip › SUPPLEMENTAL FIGURE 3.tif]
